# *Interaction Analysis* using the Cambridge Structural Database – rapid access to intermolecular hydrogen-bond frequencies and uses for coformer selection

**DOI:** 10.1107/S2052520626005135

**Published:** 2026-06-22

**Authors:** Joanna S. Stevens, Andrew G. P. Maloney, Elna Pidcock

**Affiliations:** a The Cambridge Crystallographic Data Centre 12 Union Road Cambridge CB2 1EZ UK; University of Vienna, Austria

**Keywords:** coformer selection, Cambridge Structural Database, hydrogen-bond interactions

## Abstract

A new methodology to perform virtual coformer screening, utilising data gathered on hydrogen-bonding interactions present in the organic subset of the Cambridge Structural Database (CSD), is presented. From a target molecule, commonly interacting functional groups are identified from the CSD and appropriate coformers, containing those functional groups, are suggested. The method, available as a script in *Mercury*, provides a quick and data-driven approach to coformer selection.

## Abstract 2

We describe a coformer selection tool that uses knowledge of functional group interactions found in the Cambridge Structural Database to quickly identify complementary molecules to the target.

## 1. Introduction

The Cambridge Structural Database (CSD; Groom *et al.*, 2016 ▸) is a database of small molecule organic and metal–organic crystal structures, now totalling over 1.4 million entries. The CSD was created in order that the basic tenets of structural chemistry be discoverable from the study of many thousands of crystal structures. A great deal of progress in structural chemistry, from the fundamentals of molecular structure and geometry, understanding the geometries of intermolecular interactions, the design of new materials such as drugs, dyes, energetic materials and porous materials, has advanced through the analysis of datasets of crystal structures (Taylor & Wood, 2019 ▸). Continuing in this vein, we present the Interaction Database, a collection of data extracted from hydrogen-bond interactions observed in the organic subset of the CSD: donor and acceptor groups, counts of the number of observations and frequency of occurrence calculations. These data are used by the ‘*Interaction Analysis*’ methodology to identify complementary functional groups to those present in a target molecule, which forms the basis of a simple, knowledge-based approach to choosing coformers for a target molecule.

Co-crystals are of particular interest to the pharmaceutical and agrochemical sectors as a possible route to modifying the physical properties of crystalline materials containing an active ingredient (Kavanagh *et al.*, 2019 ▸; Ammar *et al.*, 2025 ▸). For example, the prevalence of new drugs in biopharmaceutics classification system Class II (Amidon *et al.*, 1995 ▸) highlights challenges with low solubility in medicines development. Co-crystallisation of an active ingredient with a pharmaceutically acceptable coformer can offer enhanced solubility profiles, which may have the desirable consequence of increasing the bioavailability of the active ingredient (Bolla *et al.*, 2022 ▸). Other important material attributes, such as mechanical properties, can also be modified through co-crystallisation (Sanphui *et al.*, 2015 ▸). Combination products, such as Entresto (a salt co-crystal of the active ingredients valsartan and sacubitril) (Shi *et al.*, 2018 ▸) can demonstrate enhanced therapeutic effects.

There are many methods available for choosing coformers virtually, developed in order to help narrow the potentially vast search space that is explored through experimental work. Examples of existing virtual coformer screening methods use consideration of common hydrogen-bond synthons (Desiraju, 2013 ▸), machine learning methods trained on datasets of aromatic compounds (Vriza *et al.*, 2021 ▸), networks of related structures in the CSD (Devogelaer *et al.*, 2019 ▸), energetic considerations and solvation models (Klamt, 2011 ▸). Here we automate a methodology that is commonly used by experimentalists: that of selecting coformers based on complementary functional groups to those in the target molecule, but rather than relying on experience and intuition as the source of knowledge, we utilise the CSD.

## 2. Methods

A python script was written, using the CSD Python API (Sykes *et al.*, 2024 ▸), to iterate over structures in the Cambridge Structural Database (2025.1, July 2025) that are present in the ‘best representative’ *R*-factor list (van de Streek, 2006 ▸). This best representative *R*-factor list contains 917290 entries, and for structures where there are multiple determinations, the structure determination with the best *R*-factor is retained. Only structures which are organic, have 3D coordinates present, are not polymeric, and which have at least a single hydrogen-bond donor (*D*) and a single hydrogen-bond acceptor (*A*) atom (*i.e.* those that are capable of forming a hydrogen bond) were considered. Aromatic and delocalised bonds were standardised, and missing hydrogen atoms were added, if necessary. Each entry was interrogated to analyse any *intermolecular* donor–acceptor interactions present in the structure. Functional groups were identified using the library of 360 definitions developed for the hydrogen-bond propensity tool (which is included in the *CSD-Materials* software package). Constraints to hydrogen-bond geometries of *D*—H⋯*A* angle ≥ 120° and H⋯*A* distance < vdW radii + 0.0 Å were applied. In addition, all possible permutations of interactions between the donor and acceptor groups present in the structure were generated and recorded. Once all structures were processed in this way, counts were made of observed *D*⋯*A* interactions and of possible but unobserved interactions as determined from the permutations. This allowed the calculation of a frequency of occurrence (FoO) for donor–acceptor pairings:
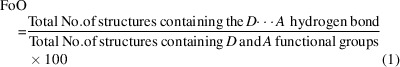
The frequency of occurrence value is interpreted as an approximation to the strength of an interaction based on the assumption that strong, energetically favourable interactions will occur frequently, and in preference to less favourable interactions. Thus a high frequency of occurrence for an interaction indicates it is seen often (when possible), *i.e.* it is a *structure-directing* interaction. There will, however, exist some biases from structures in the CSD when molecules have common combinations of functional groups (sugar moieties, for example) and these biases will be present in the frequency of occurrence values. Therefore, caution should be exercised in the interpretation of frequency of occurrence values as a definitive ranking of strength of interaction alone, particularly when the counts of interactions are low.

The consequent Interaction Database lists the hydrogen-bond donor and hydrogen-bond acceptor functional group, the total number of hydrogen bonds observed for the *D*–*A* pair, the total number of structures which contain an hydrogen bond between the *D*–*A* pair, and the frequency of occurrence. These data are broken down over structures with only a single chemical entity in the structure (number of chemical entities, NCE = 1) and those with more than one chemical entity in the structure (NCE > 1). Thus, data are gathered for interactions found in ‘pure’ forms and multicomponent forms such as solvates, salts and co-crystals.

For each donor functional group, it is therefore simple to use the Interaction Database to establish commonly observed acceptor groups and *vice versa*. In the context of co-crystal design, the *Interaction Analysis* script uses the Interaction Database to identify coformers which have functional groups that are seen to commonly interact with the functional groups present in the target molecule. Thus, the functional groups present in the target molecule are identified and the Inter­action database is queried to identify the top ten (by default) interacting functional groups with those present in the target molecule. Acceptors are identified for the target molecule donor groups, and donors are identified for the target molecule acceptor groups. The functional groups present on the molecules of the supplied coformer library are identified, and if any of these functional groups correspond to commonly observed complementary groups to the target molecule (from the Interaction Database), the coformer is listed. Thus, coformers are suggested on the basis of commonly observed interactions in the CSD. Data are presented from NCE > 1 and NCE = 1 datasets, allowing the user to choose to filter on only multicomponent data or pure form data or both.

A threshold can be set for the number of observations that are required in order for the functional group to be suggested as ‘complementary’ (defaults are 50 for NCE > 1 data and 10 for NCE = 1 data) and the user can choose how many complementary functional groups are listed per target functional group (default = 10). These defaults were selected in order to ensure a reasonable number of potential hits are returned – enough to provide a reasonable basis for screening, but not too many as to be impractical for a user. Coformers are listed with the count of observations for the interaction and the frequency of occurrence of observed interactions.

The Interaction Database contains 227492 structures and 620835 interactions, and the *Interaction Analysis* script used to query this and identify probable interactions for a target molecule only takes seconds to run and generate a report.

## 3. Results and discussion

The Interaction Database, a record of observed intermolecular hydrogen-bond interactions in the organic structures of those included in the best representative (*R*-factor) list of the CSD, has been created. The database records the donor and acceptor group identities in addition to the number of structures the interaction is observed in, along with the frequency of occurrence of interaction [equation (1)]. Therefore, included in the Interaction Database is the count of observations of an interaction, along with how commonly it is observed in systems where the relevant functional groups are present. The frequency of occurrence calculation captures information about the strength of an interaction: a high frequency of occurrence suggests a strongly favourable interaction. These descriptors are presented for all structures, for structures where there is a single chemical entity in the structure (pure forms, NCE = 1), and for structures where there is more than one chemical entity (NCE > 1) in the structure.

The combination of the Interaction Database with the *Interaction Analysis* script to query the database allows a great deal of flexibility in how this knowledge bank of interactions can be used. Here, we describe a script which focuses on the identification of coformers for a target molecule. The *Interaction Analysis* script automates the identification of likely interactions for the functional groups of the target molecule and presents the coformers (taken from the input library) which contain functional groups complementary to those in the target molecule. Details of the probable interactions are also provided in the output to allow the user to assess the frequencies of occurrence for interactions reported and understand how many interactions in the database contributed to the identification.

The *Interaction Analysis* script was designed with coformer identification as the use-case, but the script is versatile and can be used in many scenarios. Instead of providing a coformer library as the source of complementary functional groups (to those in the target molecule), a collection of counterions, solvents or any set of molecules can be provided. There is no restriction on the size of library submitted, allowing likely interactions for a pure form to be investigated in isolation (target molecule and library molecule are the same), for example.

### 3.1. Examples of use

Illustrations of knowledge which can be extracted from the Interaction Database are provided below, along with an example of using the *Interaction Analysis* script to perform a virtual coformer screen.

A simple demonstration of the knowledge contained in the Interaction Database is provided by extracting the most common donor and acceptor functional groups which participate in intermolecular hydrogen-bond interactions, along with the FoO for the interactions. Unsurprisingly, the most common interactions present are dominated by self–self interactions between functional groups which have donor and acceptor capability. Table 1 ▸ details the 10 most common interactions of which seven are self–self interactions, one is a hetero interaction between charged functional groups, and two are hetero interactions involving water either accepting from a neutral functional group or donating to a charged species. All interactions tabulated have over 2000 observations in the Interaction Database. The frequencies of occurrence for these interactions are also given in Table 1 ▸, with values ranging from 10.3 % (aromatic hydroxyl donating to aromatic hydroxyl, Donor ID: ar_oh, Acceptor ID: ar_oh) to 94.8% (NH_3_^+^ donating to carboxylate, Donor ID acyclic_T4C_nh3, Acceptor ID carboxylate_2). Diagrams of functional groups are also included in Table 1 ▸. These data highlight the difference in information encapsulated by the frequency of occurrence compared to the number observations, underscoring the point that commonly seen interactions are not necessarily strong interactions.

In multicomponent structures, many of the well-represented interactions between the different chemical entities that have very high FoO are between charged species: ammonium to sulfonato groups, carboxylate groups or halide ions, for example. There are also many high FoO interactions between neutral and charged species, such as acyclic primary amine interacting with an aromatic sulfonato (FoO 99.4%, 343 observations). The two top interactions between neutral species with the highest FoO values are aliphatic COOH or aromatic COOH donation to an aromatic nitrogen with an FoO of 94.2% and 580 observations, and 89.3% and 861 observations, respectively. The FoO values for self–self interactions between aliphatic COOH groups and aromatic COOH groups in multicomponent structures are 11.1% and 17.7%, respectively, indicating acid–acid interactions are perhaps surprisingly easily disrupted in co-crystals. The functional group with the highest FoO value for a self–self interaction in multicomponent structures is thiourea, with an FoO of 83.0% and 137 observations. Interactions where water donates to a charged anion are common amongst the high FoO value (> 80%) interactions, in agreement with the published observation that the proportion of salt structures that are hydrated is higher than the proportion of neutral structures that include water.

**Figure 1:**
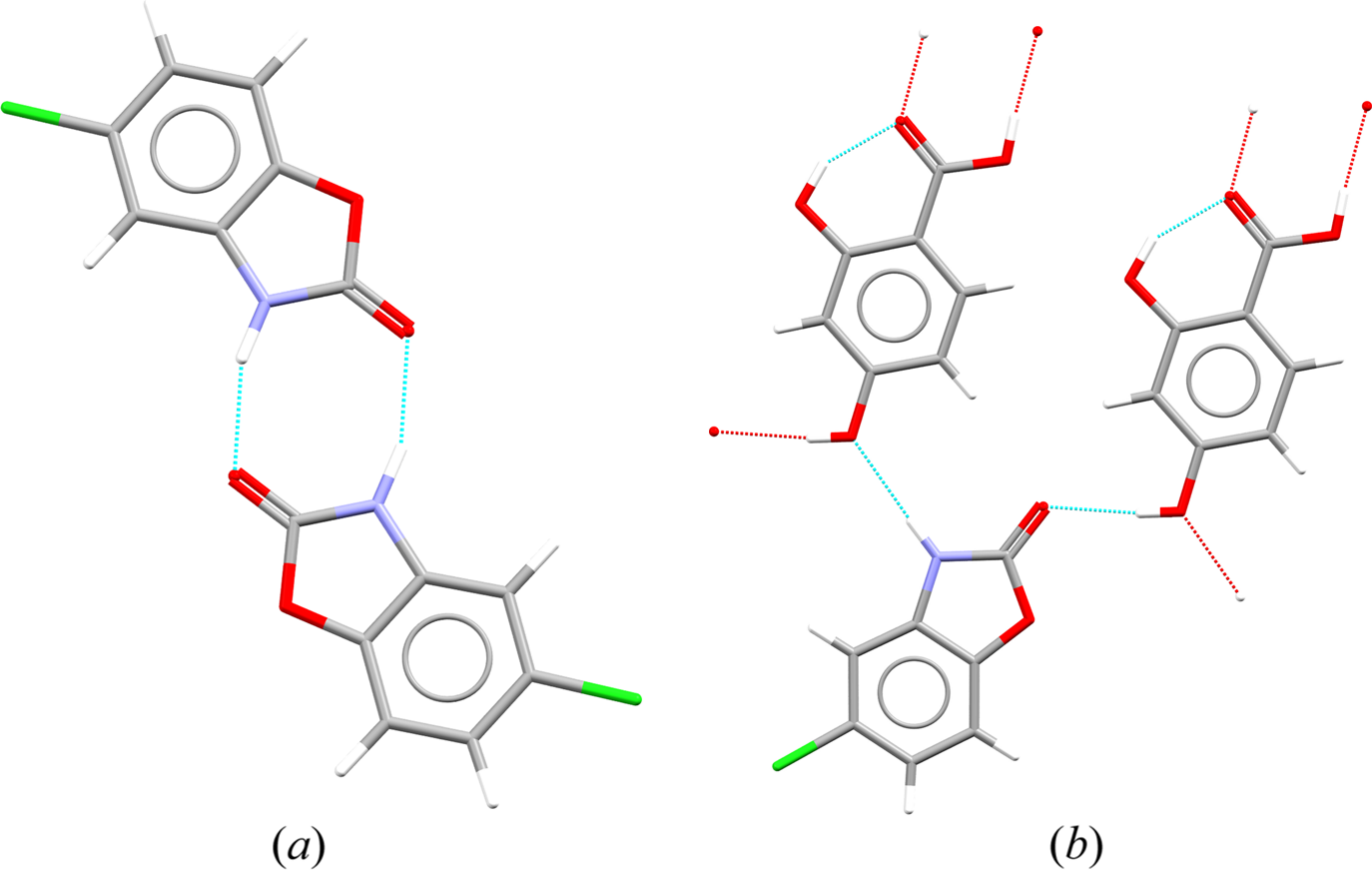
(*a*) A self–self cyclic_NH1_COOR interaction, found in pure form NEWKOP. (*b*) The self–self interaction is disrupted by the dihydroxy­benzoic acid coformer in GIDLUB. Carbon atoms are grey, hydrogen white, nitrogen blue, oxygen red and chlorine green.

Building on the observation that acid–acid interactions appear to be easily disrupted in multicomponent systems, the Interaction Database can be queried for interactions with a high FoO (> 70%) in pure forms and a low FoO (< 30%) in multicomponent forms. A minimum number of 100 structures containing the interaction of interest was applied (to NCE = 1 data) in order to have some confidence in the FoO reported for the pure form interactions. Application of these criteria return only six interactions, which are detailed in Table 2 ▸. Two self–self interactions are identified as disruptable: cyclic_amide interactions, and cyclic_NH1_COOR interactions. An example of the cyclic_NH1_COOR interaction in the pure form is found in CSD refcode NEWKOP (Ide & Topach, 1997 ▸), which is then disrupted in a multicomponent system (refcode GIDLUB; Childs & Hardcastle, 2007 ▸) where the OH groups of the dihydroxy­benzoic acid act as a donor and acceptor to the cyclic_NH1_COOR group (Fig. 1 ▸).

**Figure 2:**
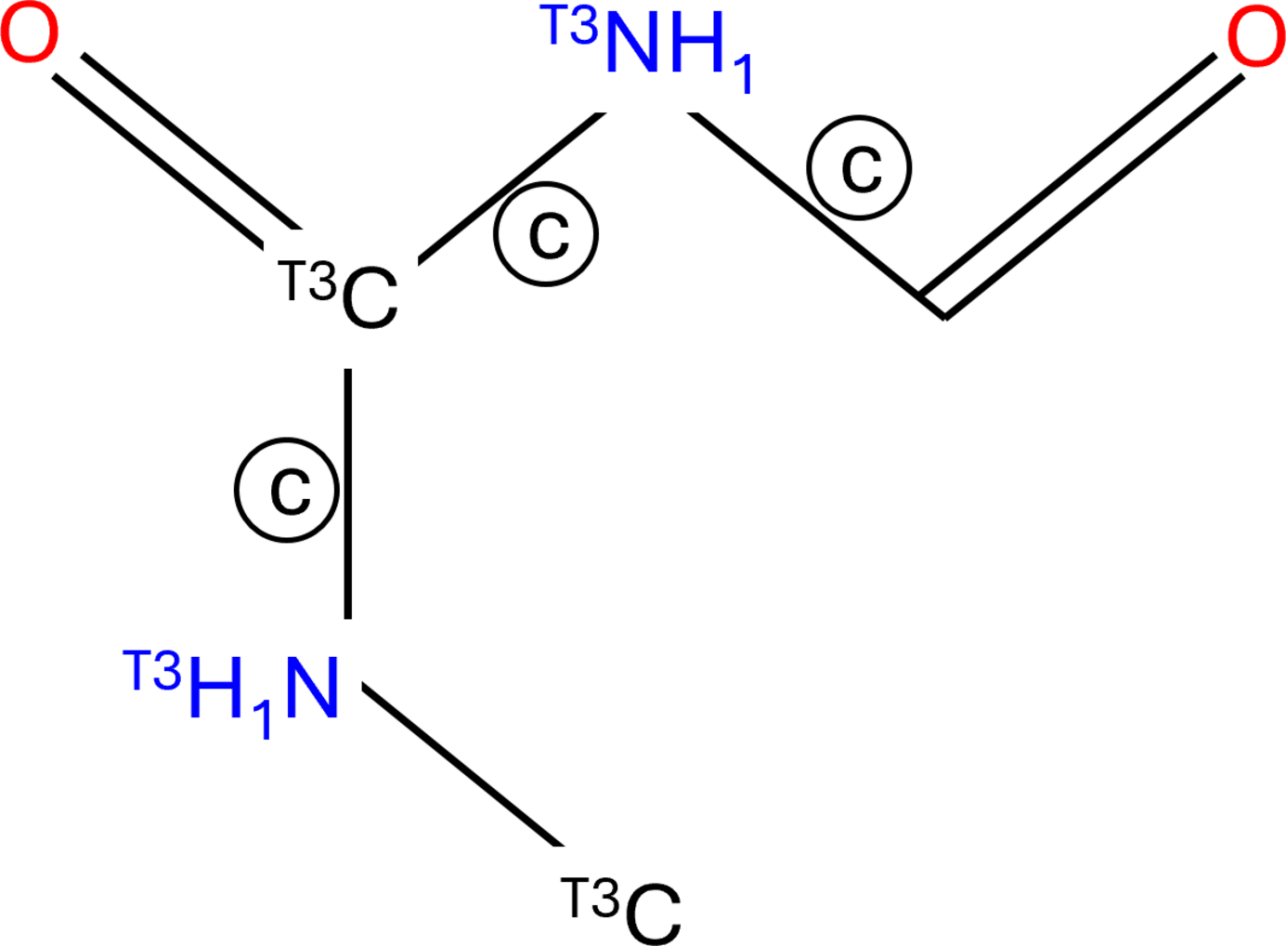
The functional group for which self–self interactions return a high FoO in multicomponent structures. T3 indicates the atom is bonded to three atoms, and an encircled ‘c’ indicates the bond is cyclic.

To identify the least disruptable interactions, FoO values for both the pure form and the multicomponent form greater than 70% were chosen (a minimum number of 50 structures containing the interaction of interest in the pure form was applied). Ten interactions were identified and five of these involve donations to an anionic species: four to carboxylate and one to phosphonate. The urea-containing functional group, shown in Fig. 2 ▸, participates in the only self–self interaction that meets the above criteria.

The above examples represent a few snapshots of knowledge which can be gleaned from the Interaction Database. However, as mentioned above, the main reason for building the database was to develop a coformer screening tool which utilised the Interaction Database. The following example illustrates a coformer screening workflow for the target molecule paracetamol using the default library of coformers supplied as part of the *CSD-Materials* software package. Paracetamol was selected from the structure navigator in *Mercury* (Macrae *et al.*, 2020 ▸) using the refcode HXACAN (Haisa *et al.*, 1974 ▸). The *Interaction Analysis* script was run from the *CSD Python API* menu, under the sub-menu *Prototypes*. The script uses the current structure as the target molecule. The user may choose a coformer library, the number of target complementary functional groups (in this example, 30), and the number of structures required in the Interaction Database in order to recommend an interaction. Alternatively, the user may accept the default settings offered by the dialog as outlined in Section 2. When the script has completed, an html version of a summary report is presented, and a .docx file is written to the output directory. Coformers that contain complementary functional groups to the target molecule using interactions from NCE > 1 structures and NCE = 1 are presented first along with the highest FoO and number of the possible *D*⋯*A* interactions that relate the two molecules. Thus the user is able to choose possible coformers based on the ‘strength’ of the possible interaction (using the FoO) or the number of potential interactions available to the target coformer pair. The standard coformer library, released with *Mercury*, was used and an example of the output is shown in Table 3 ▸.

Also included in the output are the functional groups identified in the target molecule, separated into donors and acceptors. In the case of paracetamol, two functional groups are identified: ar_al_trans_amide (amide attached to both aromatic carbon and aliphatic saturated carbon) and ar_oh (aromatic hydroxyl), see supporting information for details. The next tables in the report detail the complementary acceptor groups to the target donors found in NCE > 1 structures, followed by those found in NCE = 1 structures. Included in the tables are fields taken from the Interaction Database: the FoO for the interaction, and the number of structures along with the name of any coformer (in the library) that contains the complementary functional group. These tables are useful for understanding which functional group interactions contribute to the total number of possible interactions between the target molecule and coformer listed earlier. Similar data for the acceptors of the target molecule are also presented.

In Table 3 ▸, lactobionic acid is found to have four possible interactions with paracetamol. These interactions are between the aromatic hydroxyl (ar_oh) group and the two chemically different hydroxy groups of lactobionic acid: the target ar_oh group can act as the donor to or acceptor of such interactions, hence a total of four possible interactions. The highest FoO of 35.4% represents a mid-range value, which may suggest a reasonable hydrogen-bond-based driving force for co-crystallisation. A coformer with a higher FoO is nicotinamide, which has two possible interactions, involving donation from the target molecule’s ar_oh group to an aromatic nitrogen (aromatic_N, FoO 71.6%) or to a carbamoyl C=O (carbamoyl_2, FoO 28.0%). Whilst there is no crystal structure of a paracetamol nicotinamide co-crystal in the CSD, paracetamol does crystallise with pyridine, displaying an OH to aromatic N interaction. The amide group of paracetamol has only three complementary groups that pass the criteria for inclusion from the NCE > 1 data: itself, Cl^−^, and water. Given the lack of data from multicomponent structures, the user may wish to draw on observations from NCE = 1 structures where more functional groups, for example, cyano, amides, and acids, pass the criteria.

In order to explore the efficacy of *Interaction Analysis* for co-crystal screening, 13 coformers from paracetamol co-crystal structures in the CSD were chosen on the basis of the variety of functional groups present for screening by the *Interaction Analysis* script. The target number of complementary interactions was increased from the default of ten, to 30, and the results for the 13 coformers are given in Table 4 ▸. Eight of the 13 coformers were identified as possible candidates from consideration of the NCE > 1 data by the *Interaction Analysis* script. Five of the eight coformers return a value for the highest FoO interaction above 30% and seven have a value for the highest FoO above 20%. The observed coformers have one or two possible interactions identified, and the highest FoO interactions are all donations from the aromatic hydroxyl (ar_oh) group of paracetamol to a coformer acceptor group, except in the case of picric acid where the interaction is donation from ar_oh of picric acid to ar_oh of paracetamol. In seven of the coformers, the interaction with the highest FoO value is observed in the crystal structure. It is noted that a co-crystal is obtained with picric acid which has a value for the highest FoO interaction of just 12.1%. Therefore, as with many virtual coformer screening tools, *Interaction Analysis* provides guidance for choosing coformers utilising knowledge of likely interactions, but does not capture all the nuances governing how different chemical entities crystallise together.

As mentioned above, there are few observations for the ar_al_trans_amide group from the NCE > 1 (multi-component) dataset, and hence coformer suggestions in Table 4 ▸ are only based on interactions with the hydroxy group of paracetamol. The NCE = 1 (single component) dataset identified likely interactions from and to the ar_al_trans_amide group of paracetamol and so increased the number of possible interactions between the target molecule and coformer, and identified a further two coformers: diaminocyclohexane and tetracyanoquinodimethane. Thus, both sets of results, from the NCE > 1 and NCE = 1 datasets were useful: the NCE = 1 dataset provided more information about the interactions involving the trans amide group, supported results from the multicomponent structures, and identified additional coformers, while the NCE > 1 dataset utilised relevant information from structures which include possible disruption of target-target functional group interactions.

Finally, the output from the script includes a table of coformers with their donor functional groups listed, and a similar table where the acceptor functional groups are listed. This is a valuable resource to understand the chemical diversity present in a coformer library.

*Interaction Analysis* has been performed for a few target molecules where both positive and negative results for coformer screens have been reported across the literature. Results for paracetamol, virtually screened against 44 experimentally trialed coformers, returned 18 suggested coformers. Nine of those coformers suggested by *Interaction Analysis* were true positives, though 21 co-crystals were found experimentally. Fourteen true negatives were identified and the overall accuracy of the virtual screen was 52.2% [(9+14)/44], and 50% of suggested coformers were observed as co-crystals. An *Interaction Analysis* screen using Loratadine as the target molecule correctly suggested 17 of the 31 coformers found to form co-crystals experimentally, whilst suggesting six coformers which did not result in a co-crystal. Four of the 17 coformers returned two common interactions with Loratadine functional groups and all four were found experimentally. For Praziquantel, *Interaction Analysis* suggested that 30 coformers from a list of 34 would form co-crystals, and 16 coformers were found experimentally, yielding a good accuracy for the positive predictions (13/16 coformers were correctly identified) but a low precision. Again, 9/12 coformers that were identified with more than one common interaction with the target molecule were found experimentally. In our hands, *Interaction Analysis* has performed with modest accuracy and/or precision: the simplicity of the methodology being unlikely to result in very accurate or precise predictions. However, the tool provides a list of coformers quickly and straightforwardly, and provides some prioritization of that list, based on the number of common interactions with the target molecule.

### 3.2. Future areas of development

The functional group library utilised by default is that designed for the hydrogen-bond propensity tool. There are multiple definitions for some functional groups, for example, hydroxy groups. These differences in definitions may not be entirely appropriate for the screening of potential coformers: some aggregation of the chemical definitions would result in larger subsets of interactions, and hence more reliable frequency of occurrence values. This will be the focus of future work.

Currently the *Interaction Analysis* script identifies complementary functional groups based only on the number of observations in the Interaction Database. This is useful if the user is interested in interactions with solvents, charged, and neutral species, but the option to specify types of interaction to include or exclude would be valuable in some use-cases. An alternative mechanism of selecting complementary functional groups could include the frequency of occurrence value, either along with the number of observations or instead of the number of observations.

## 4. Conclusions

A dataset of intermolecular hydrogen bond interactions (the Interaction Database) was compiled from a large subset of the organic structures in the Cambridge Structural Database. This dataset is utilised by the *Interaction Analysis* script to identify complementary interactions with the functional groups present in a target molecule. The initial use-case for this script targeted virtual coformer screening and aimed to automate a common methodology used by experimentalists: to choose coformers based on familiar or likely interactions with the target molecule. The script has been made available as part of the 2025.3 CSD software release and can be accessed through the *CSD Python API* menu in *Mercury*. The user can use their own library of coformers by pointing the script at a directory of mol2 files, or can use the default library supplied in the software package. The coformers that are identified by the script are presented with the highest frequency of occurrence for an interaction between the target molecule and coformer, and the number of observations for the interaction found in the Interaction Database. The results are also divided into suggestions made from consideration of only multicomponent structural data (NCE > 1) or pure form data (NCE = 1). Thus the user is able to make a selection of coformers based on strength of possible interaction (frequency of occurrence), common interactions (number of observations), chemical diversity (enumeration of functional groups present in library) or a combination of all the above. The script can be used for both charged or neutral species. It is quick to run, only taking seconds to generate the output report, and provides CSD-based insights to guide coformer selection.

## Supplementary Material

Interaction Analysis script output for paracetamol (HXACAN). DOI: 10.1107/S2052520626005135/dzi5003sup1.pdf

## Figures and Tables

**Table 1:** Common interactions within the CSD Donor ID and Acceptor ID give the functional group names as listed in the hydrogen-bond propensity functional group library (included in the *Mercury* installation directory). ‘al’ signifies aliphatic, and ‘ar’ signifies aromatic. Numbers appended to functional group name (*e.g.* al_hydroxy_3) are identifiers and do not impart any chemical meaning. In the Donor and Acceptor diagram columns, atoms with a superscripted TX are defined as having X connections, encircled ‘a’ or ‘c’ signify respectively acyclic or cyclic bonds, ‘v’ indicates variable bond type.

Donor ID	Donor diagram	Acceptor ID	Acceptor diagram	Counts	FoO (%)
Water	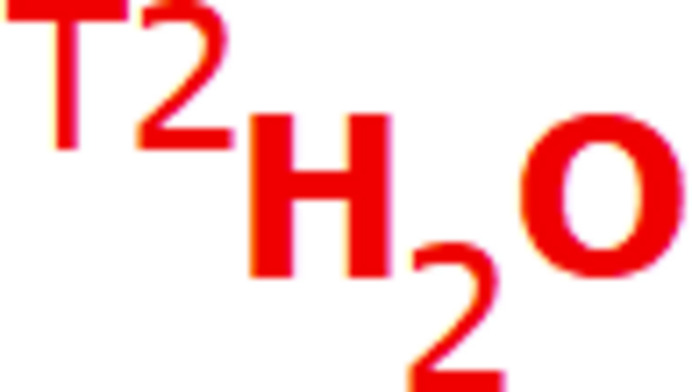	Water	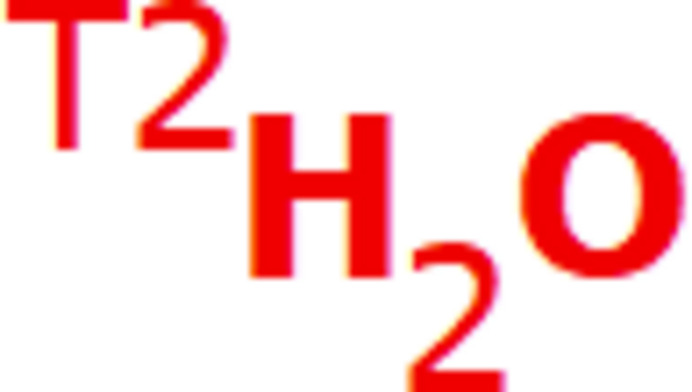	7448	26.7
al_hydroxy_3	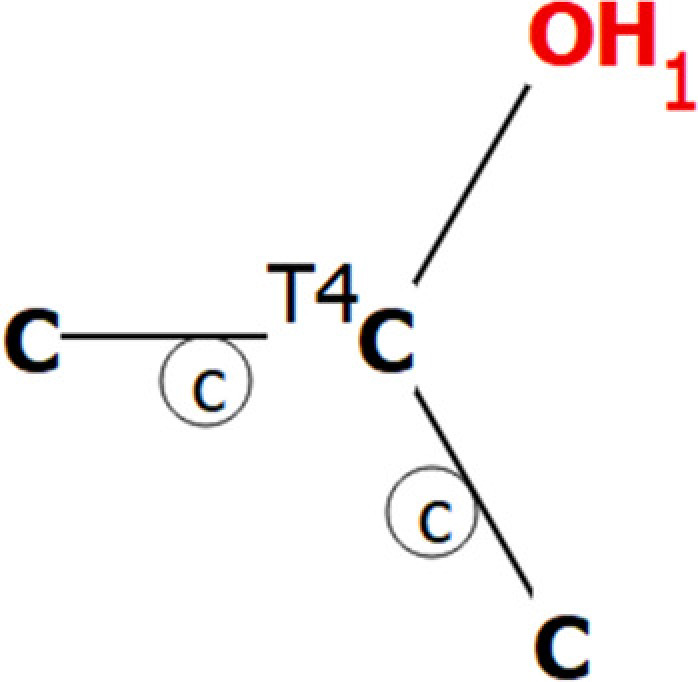	al_hydroxy_3	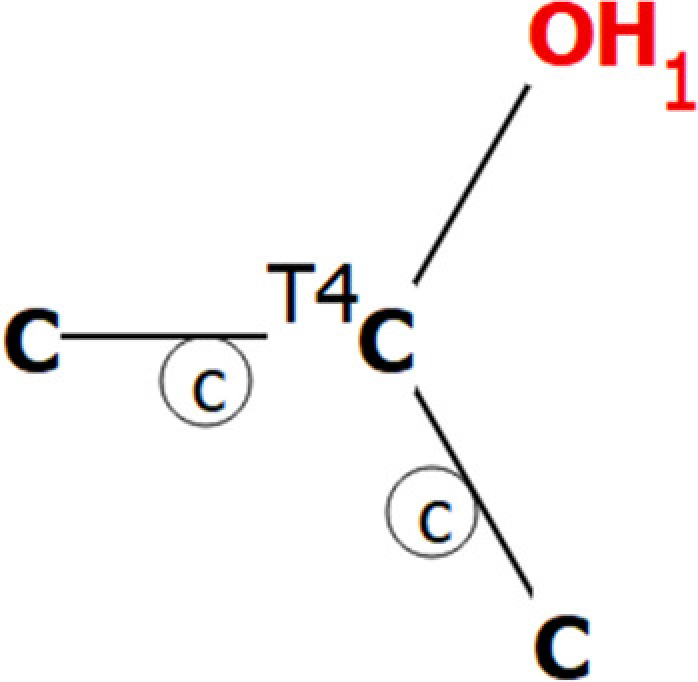	7411	26.2
al_al_amide	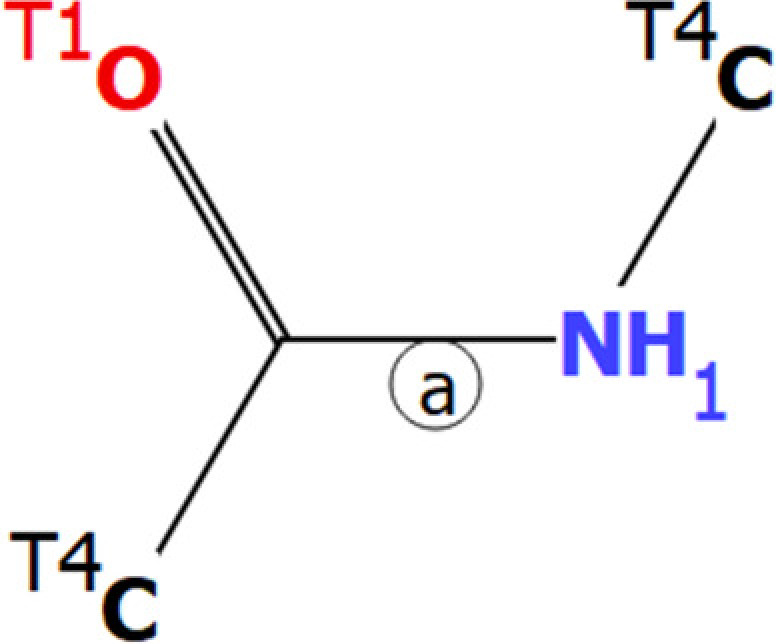	al_al_amide	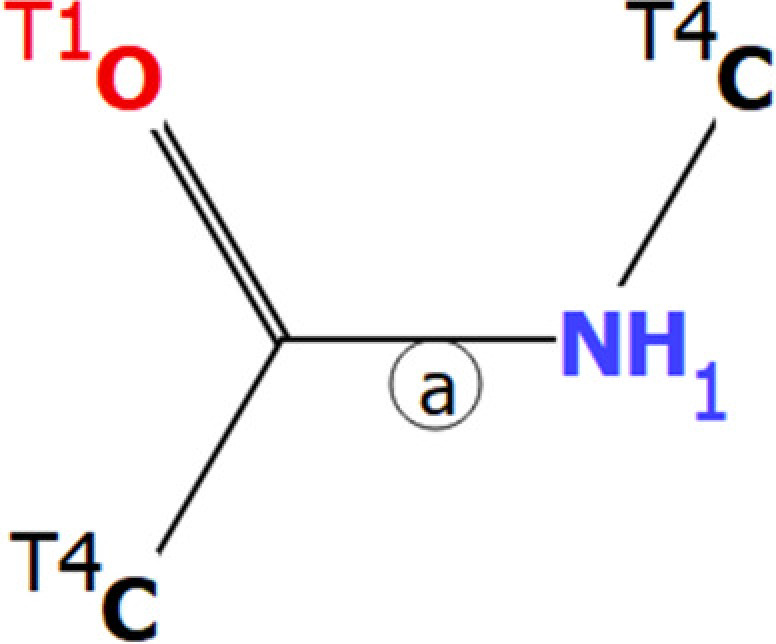	3225	43.6
al_cooh_1	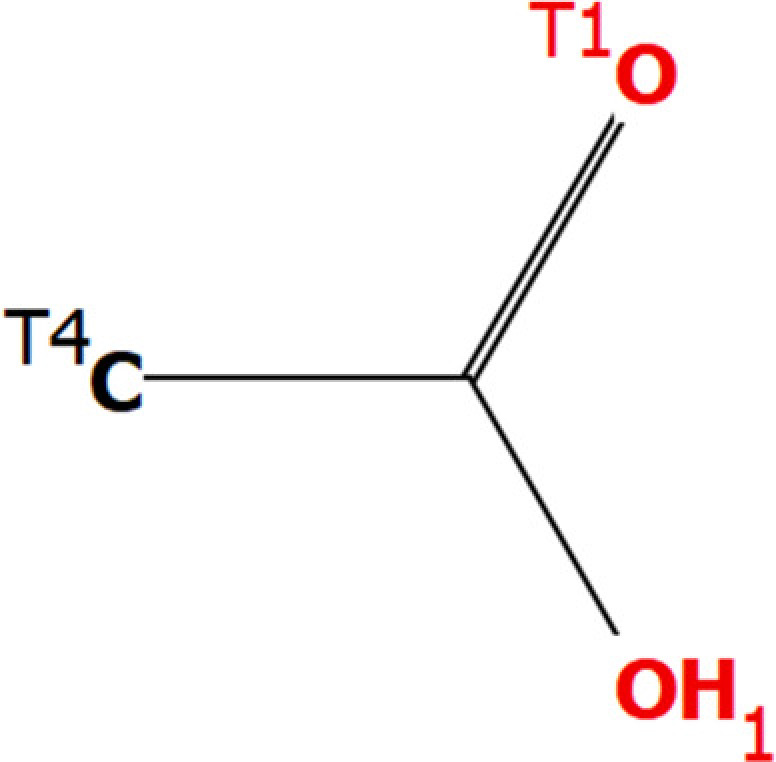	al_cooh_1	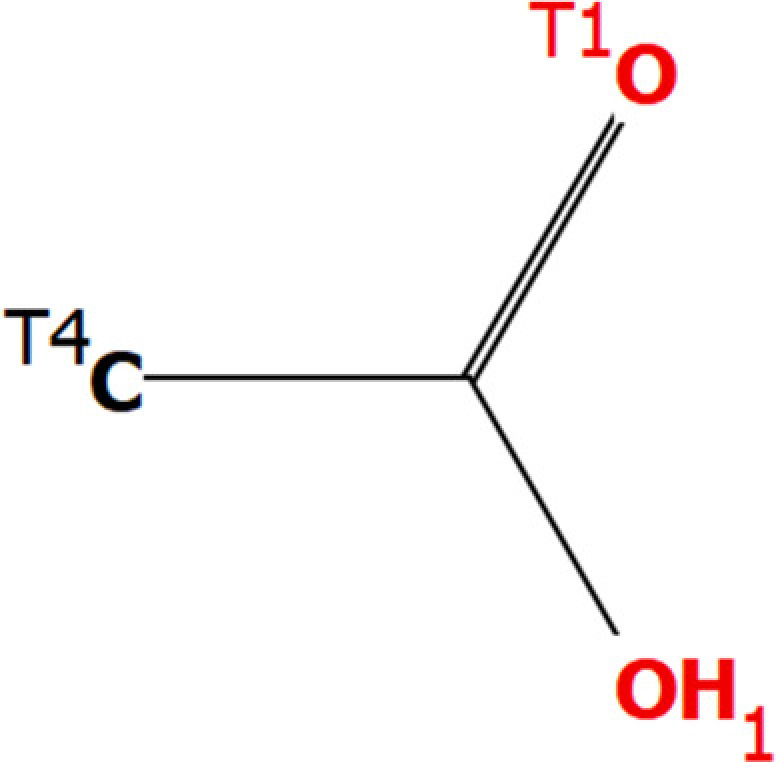	3081	28.0
ar_oh	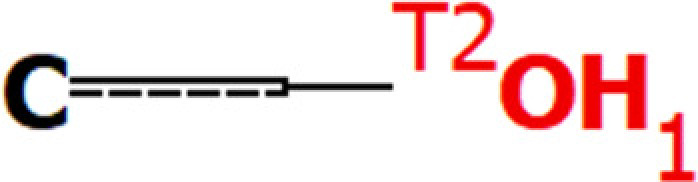	ar_oh	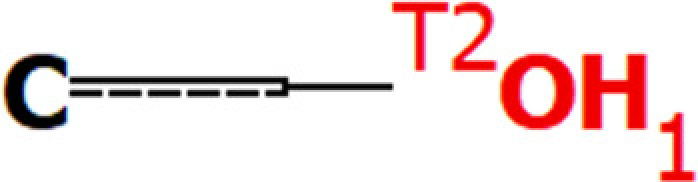	3016	10.3
acyclic_al_oh	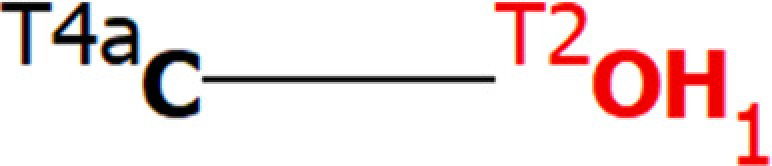	acyclic_al_oh	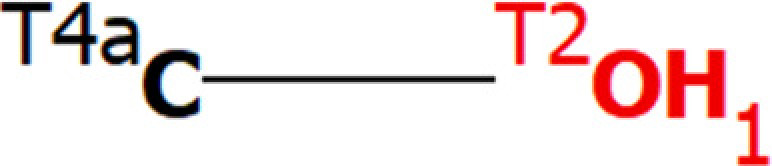	2758	16.3
ar_cooh_1	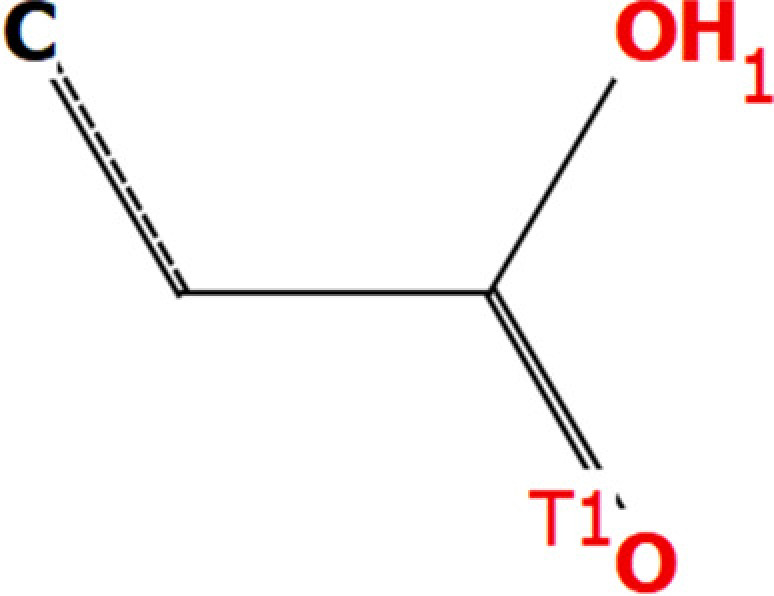	ar_cooh_1	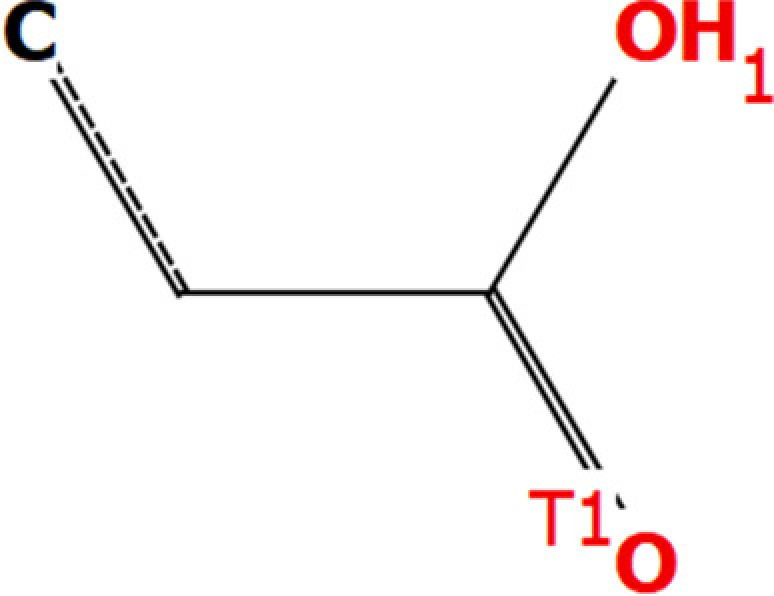	2498	32.4
al_hydroxy_3	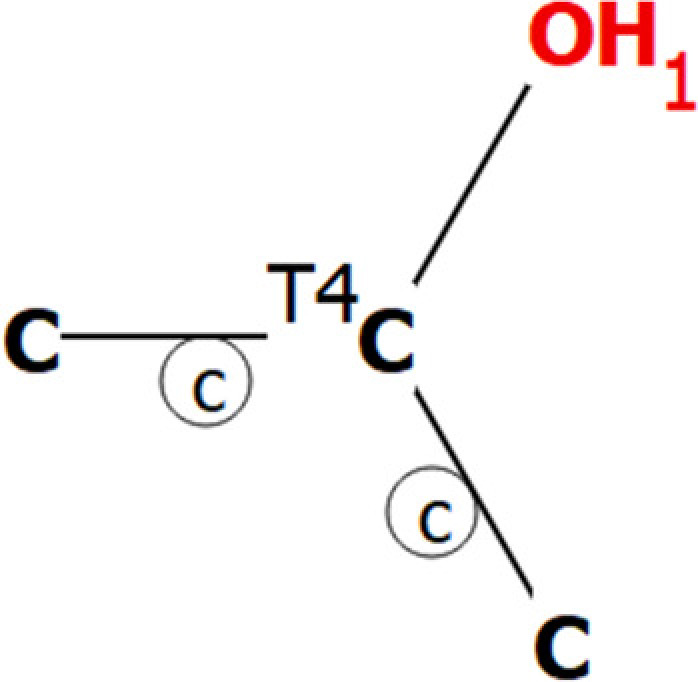	Water	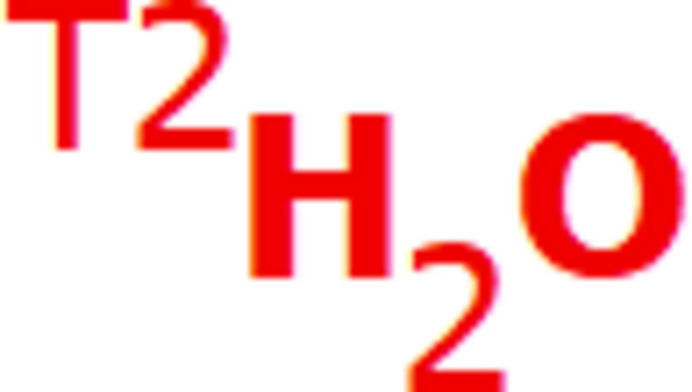	2448	64.9
Water	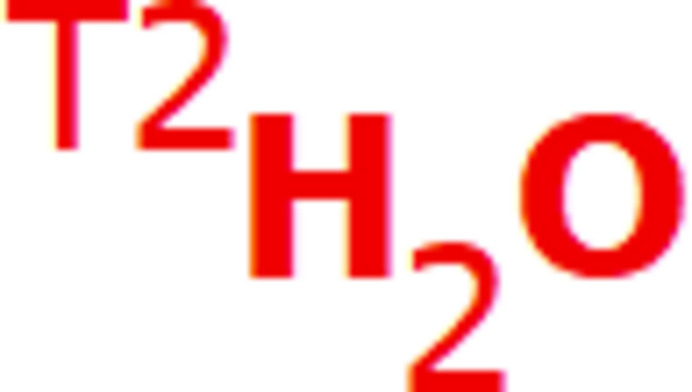	Cl^−^	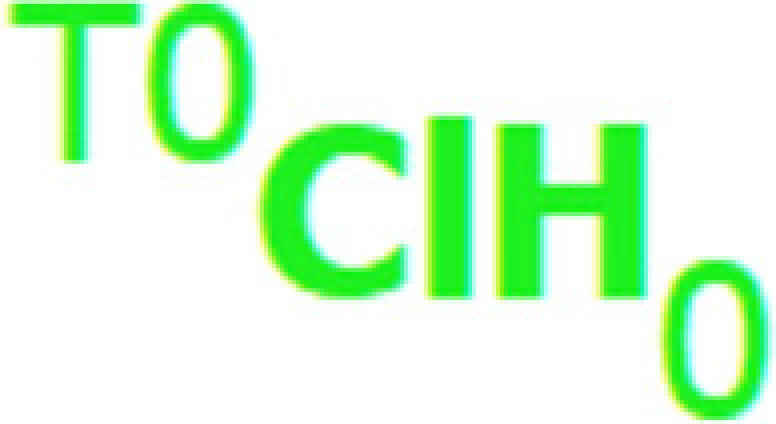	2420	91.7
acyclic_T4C_nh3	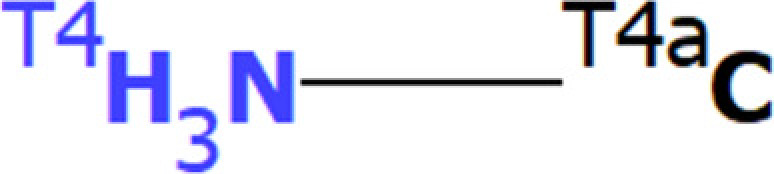	carboxylate_2	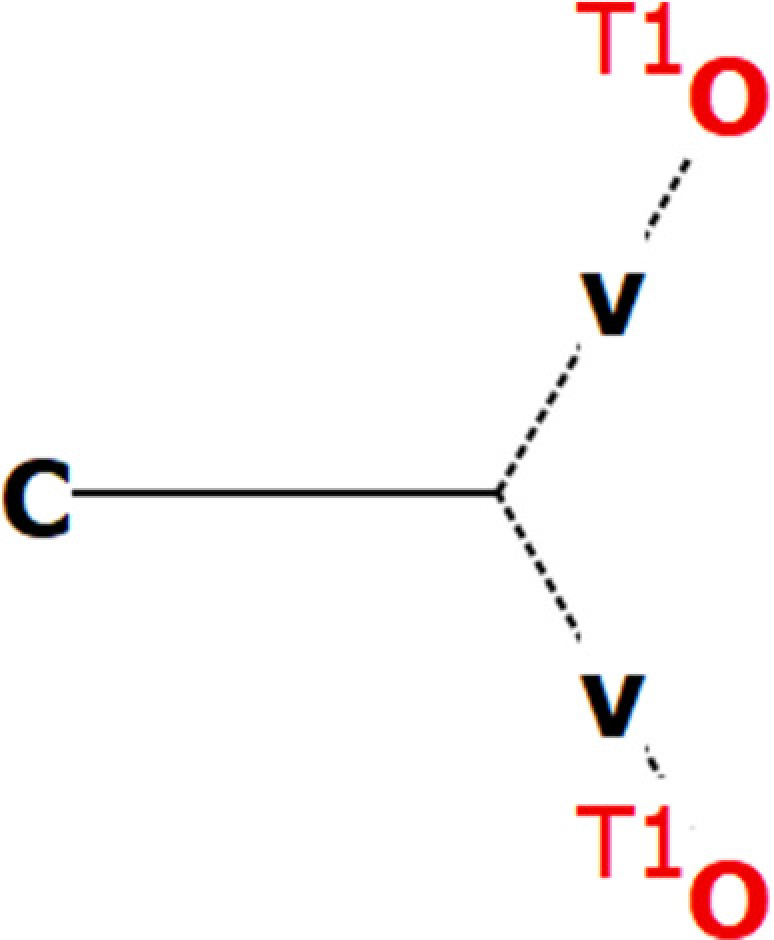	2391	94.8

**Table 2:** Donor–acceptor interactions with high and low frequency of occurrence (FoO) values in NCE = 1 and NCE > 1 structures, respectively

				FoO (%)	
Donor ID	Donor diagram	Acceptor ID	Acceptor diagram	NCE = 1	NCE > 1
phosphinic_amide	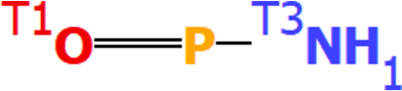	phosphonate	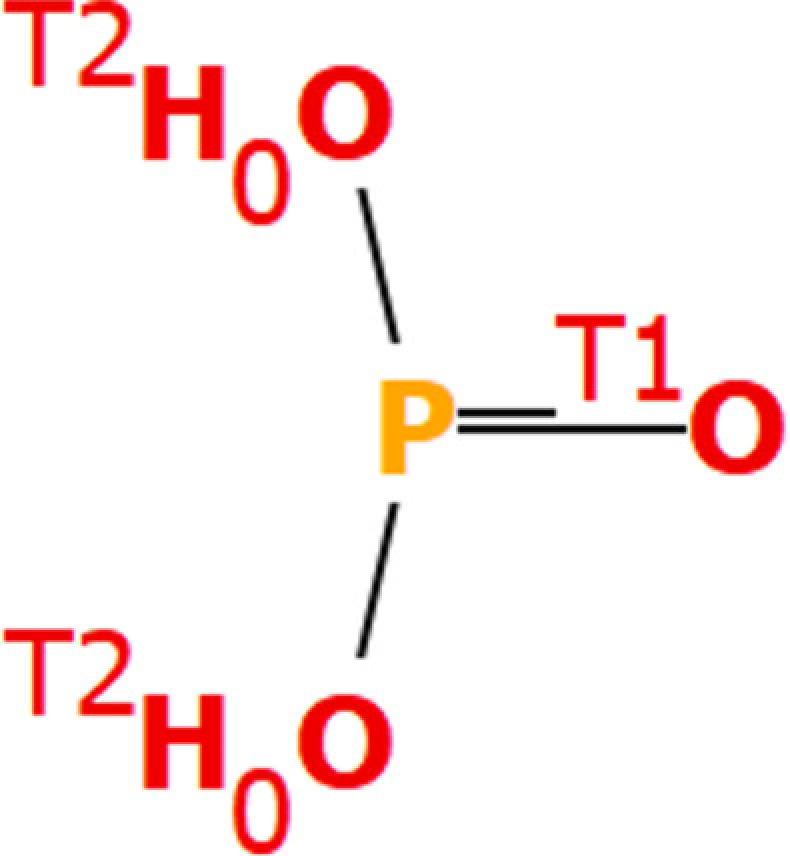	81.6	20.0
cyclic_amide	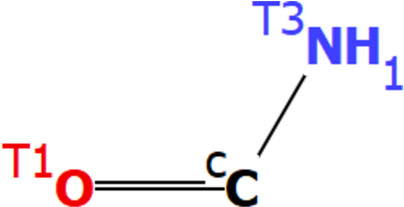	cyclic_amide	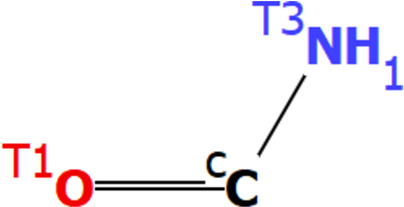	79.7	27.0
cyclic_al_oh	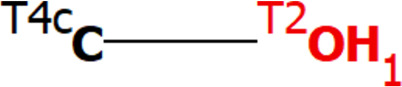	carbonyl_2	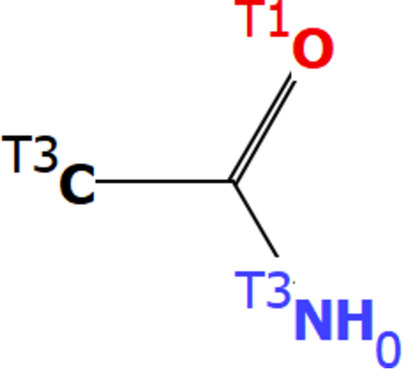	79.5	24.3
cyclic_NH1_COOR	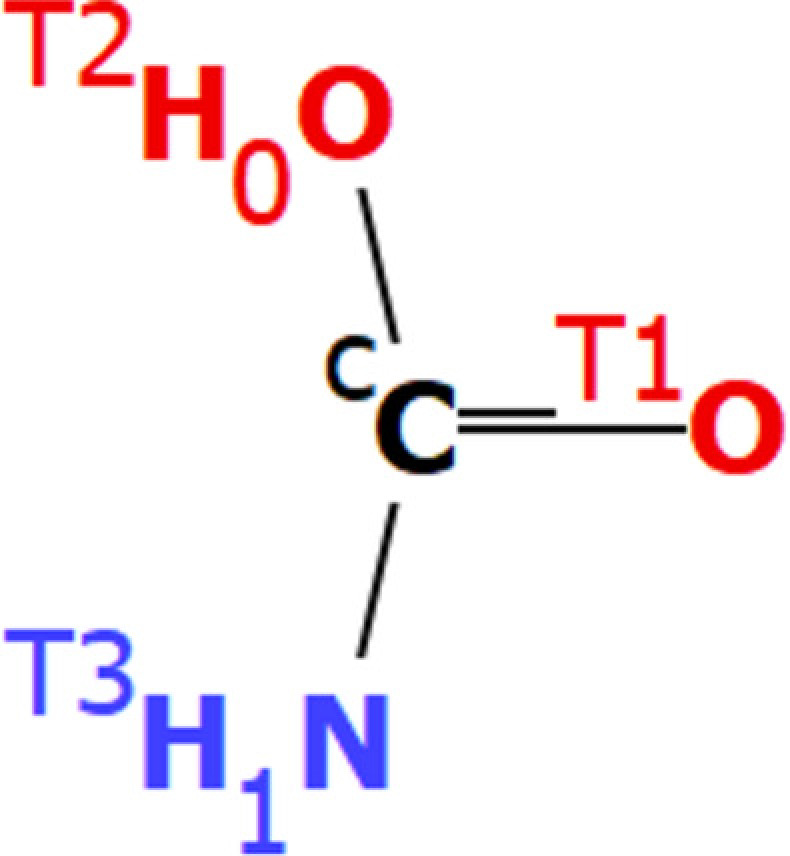	cyclic_NH1_COOR	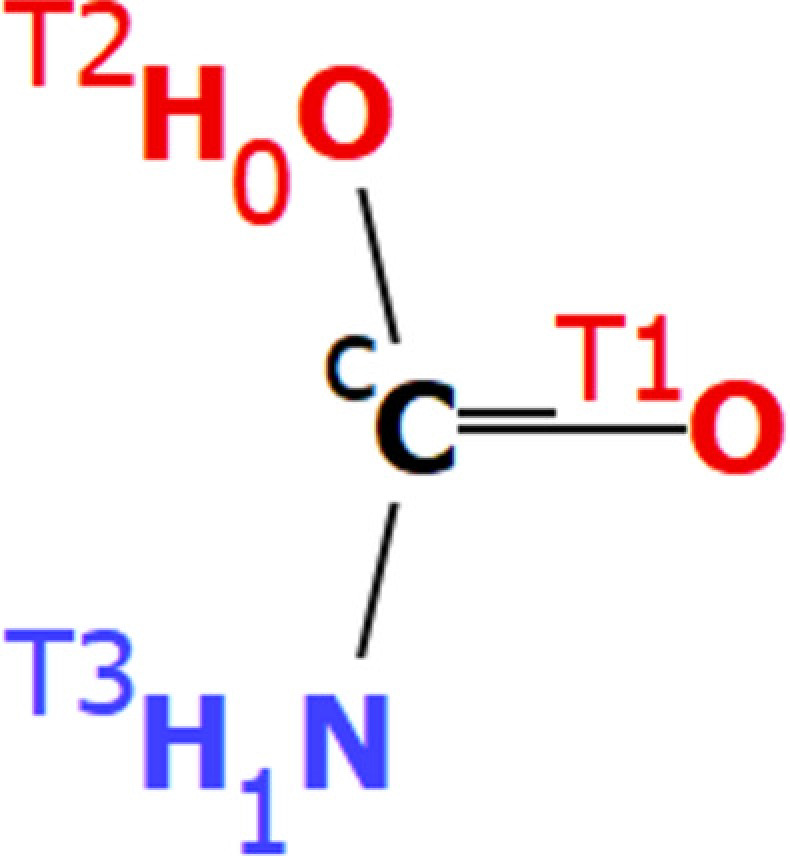	75.1	29.4
al_cooh_1	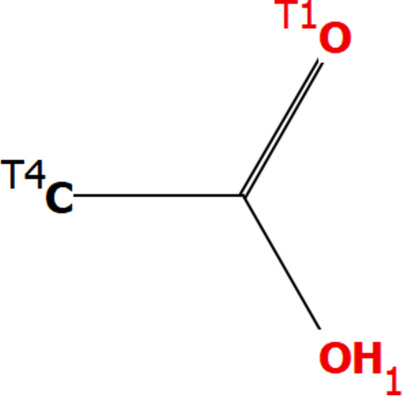	al_hydroxy_2	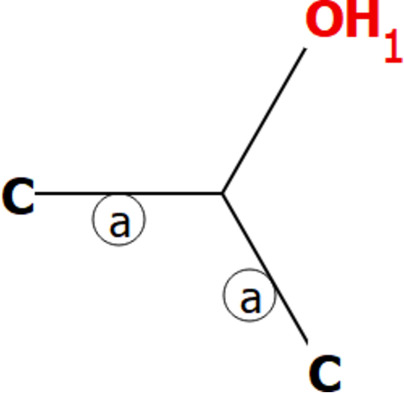	71.6	4.6
Indole	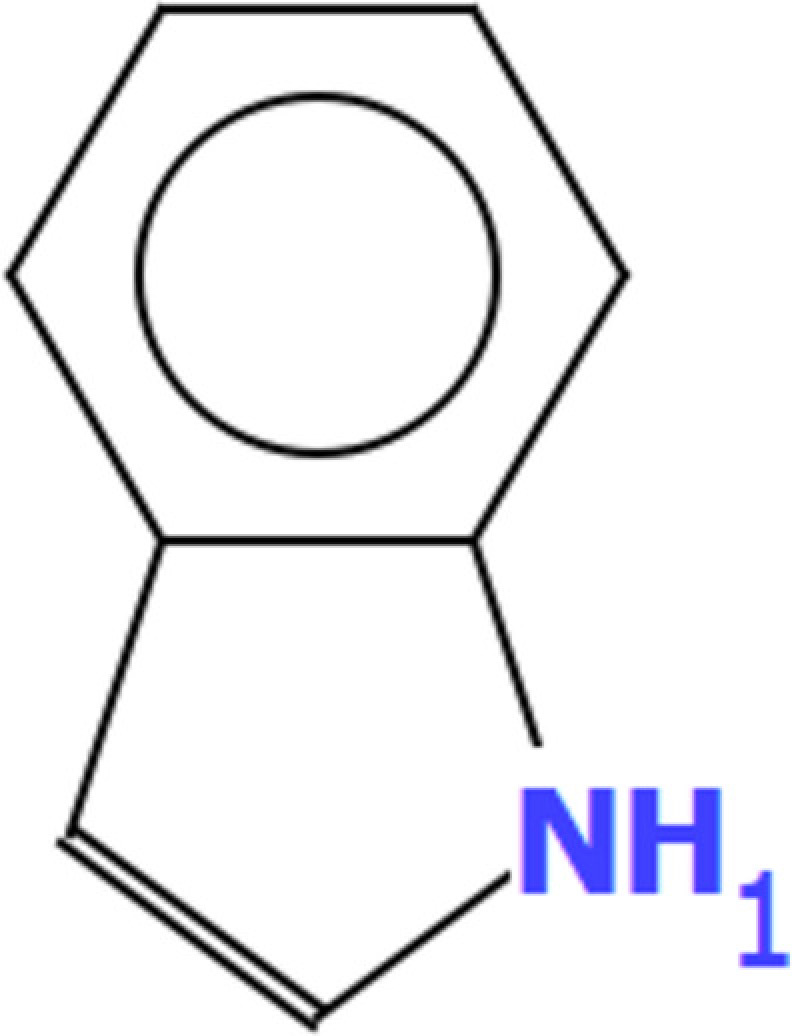	cyclic_ketone_1	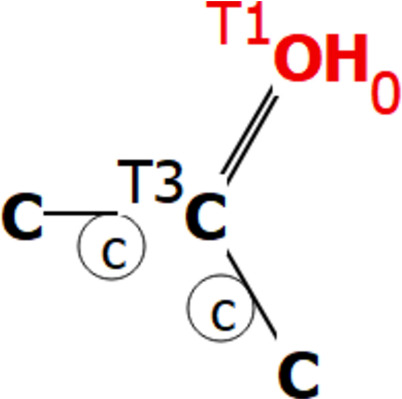	70.2	29.3

**Table 3:** Output from the *Interaction Analysis* script for the target molecule paracetamol, listing examples of chosen coformers using NCE > 1 data

Coformer	No. of possible interactions	Highest FoO found (%)
Lactobionic acid	4	35.4
Nicotinamide	3	71.6
Theophylline	2	41.4
Citric acid	2	28.1
Pyrazine	1	71.6
4-Hydroxybenzoic acid	1	12.1

**Table 4:** Thirteen coformers observed to crystallise with paracetamol, the donor and acceptor groups identified within the coformer, and the highest frequency of occurrence (FoO) value given for the possible interactions with paracetamol Groups highlighted in bold are those that are utilised in hydrogen-bonding interactions in the co-crystal structure, and which return the frequency of occurrence value listed.

Coformer	Donor ID	Acceptor ID	FoO (%)
Morpholine	saturated_ring_NH	cyclic_ether_1, **saturated_ring_NH**	52.1
Citric acid	acyclic_al_oh, al_cooh_1	**acyclic_al_oh**, al_cooh_1	28.1
Picric acid	**ar_oh**	ar_nitro, ar_oh	12.1
Maleic acid	carboxylic_acid	carboxylic_acid	–
Theophylline	imidazole_2	cyclic_amide_6, carbonyl_2, imidazole_2	41.4
Ethanedioic acid	keto_carboxylic_acid	keto_carboxylic_acid	–
Piperazine	saturated_ring_NH	**saturated_ring_NH**	52.1
Dimethylpiperazine	–	**al_tert_amine_1**	34.0
Pyridinedicarboxylic acid	ar_nh, ar_cooh_1	**ar_carboxylate**, ar_cooh_1	32.6
Diaminocyclohexane	al_prim_amine	al_prim_amine	–
Bisphenazine	–	**ar_nitrogen_2**	46.9
Dioxane	–	dioxane	–
Tetracyanoquinodimethane	–	cyano	–

## Data Availability

The output of the paracetamol coformer screen is provided as supplementary material. The *Interaction Analysis* script is available through CCDC’s *CSD-Materials* software version 2025.3.
