# Supplementary material for: Interaction Analysis using the Cambridge Structural Database – rapid access to intermolecular hydrogen-bond frequencies and uses for coformer selection
Source: Acta Crystallogr B Struct Sci Cryst Eng Mater. 2026 Jun 22;82(Pt 4):391–8. doi: 10.1107/S2052520626005135 (PMC13470796; doi:10.1107/S2052520626005135)
Supplement: Supplementary file 1 [file b-82-00391-sup1.pdf]

Acta Cryst B

STRUCTURAL SCIENCE
CRYSTAL ENGINEERING
MATERIALS

Volume 82 (2026)

Supporting information for article:

Interaction Analysis using the Cambridge Structural Database – rapid access to intermolecular hydrogen-bond frequencies and uses for coformer selection

Joanna S. Stevens, Andrew G. P. Maloney and Elna Pidcock

Interaction Analysis (IA)

Interaction informed coformer selection tool which takes the target molecule, extracts its functional groups, and returns a list of groups which are commonly seen to interact with the chemistry of the target molecule (based on frequency of occurrence considerations) to help choose suitable coformers based on common hydrogen bond interactions that are seen in the CSD (or another database if specified by the user).

1. Top coformers for HXACAN

The top ranked coformers from the selected coformer library are based on the Interaction Analysis score (IA score) – the number of matches for top interacting coformer functional groups with the target molecule. This is based on popular intermolecular hydrogen bonding for multicomponent structures in the Interaction Database (prefer to Methodology below for details). The frequency of occurrence (%) for the most popular interaction is also reported. Analogous results using popular interactions found in single component structures are shown in grey.

Top ranked coformers for HXACAN from Interaction Analysis (IA) based on popular interactions in multicomponent structures (frequency of occurrence in Interaction Database).

Coformer	IA score	Highest frequency of occurrence
KIGLUI	2	41.36 %
AMUBAM	2	28.05 %
AHEPUY	1	52.21 %
MUPPUI	1	52.21 %
LUJSOZ	1	46.85 %
MUPPIW	1	34.04 %
SUTVAF	1	32.62 %
BODSUL	1	12.09 %

Top ranked coformers for HXACAN from Interaction Analysis (IA) based on popular interactions in single component structures (frequency of occurrence in Interaction Database).

Coformer	IA score	Highest frequency of occurrence
AMUBAM	6	49.12 %
BODSUL	3	51.47 %
KIGLUI	3	47.97 %
WIGCEW	2	61.29 %
SUTVAF	1	43.48 %
RECJOC	1	32.69 %
AHEPUY	1	24.64 %
MUPPUI	1	24.64 %

LUJSOZ	1	12.7 %

Methodology

Each functional group in the target structure (HXACAN) has been checked against an intermolecular hydrogen bond database created from the CSD Best R-factor List, returning what each functional group interacts with most commonly (top 30 interactions based on frequency of occurrence) and seeing whether a corresponding acceptor/donor is present in any of the coformers in the specified coformer library:

1) Identify functional groups in the target
2) Look up the "favourite" interacting functional groups per target functional group in the Interaction Database
3) Find which coformers contain the favourite functional groups from the specified coformer library
4) Consolidate the results to give a list of coformers with the potential for one or more of the 'favourite' interactions

The results are shown above (left-hand table), obtained by checking against interactions observed for multicomponent structures in the Interaction Database (structures with more than one unique chemical component – number of unique chemical entities NCE > 1). This could be used to help choose suitable coformers based on common hydrogen bond interactions that are seen in the CSD for the target structure. Analogous results are shown by checking against interactions observed for single component structures in the Interaction Database (NCE = 1, right-hand table highlighted in grey).

The report also provides:

- Target structure functional groups & the coformer library and interaction database used (Section 2)
- Which coformer functional groups were matched against each target donor and target acceptor group (Section 3 and Section 4). This could allow specific functional groups to be targeted, or to compare frequency of occurrence for self-coformer vs. self-self interactions of specific functional group(s) using the multicomponent and single component data.
- List of all functional groups in the coformer library (Section 5).

2. Target structure, Coformer library & Interaction database

Target structure:

HXACAN

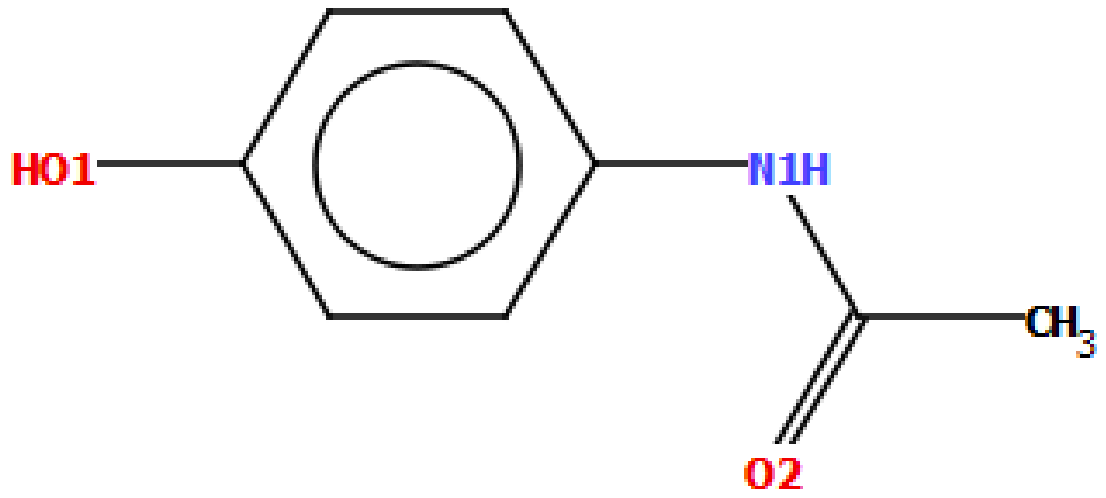

Figure 2.1. 2D diagram for HXACAN

Table 2.1. Functional groups in HXACAN

Atom label	Donors	
N1	ar_al_trans_amide	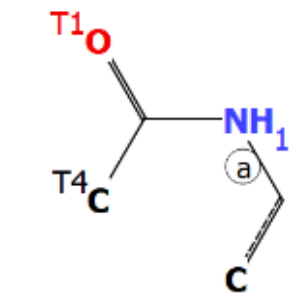
O1	ar_oh	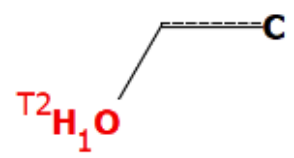

Atom label	Acceptors	
O1	ar_oh	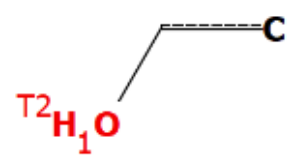
O2	ar_al_trans_amide	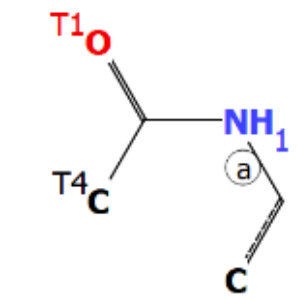

Coformer library:

D:/Papers/Mine/InteractionAnalysis/HXACAN/coformers

Coformers:

AHEPUY, AMUBAM, BODSUL, JEMTEE, KIGLUI, LUJSOZ, LUJTAM, MUPPES, MUPPIW, MUPPUI, RECJOC, SUTVAF, WIGCEW

Interaction Database:

Default CSD interaction database

(best R-factor 2025.1 July25, criteria: organic, 3D coordinates, not polymeric, at least 1 hydrogen bond donor and 1 hydrogen bond acceptor)

Top 10 interactions per donor in HXACAN

Table 2.2. Top 30 interactions per donor in HXACAN by frequency of occurrence for multicomponent database hits (NCE > 1, ≥ 50 structure threshold), and acceptor matches found in the specified coformer library.

		Multicomponent (NCE > 1)				
**H-bond Donor in HXACAN**	**Functional group definition**	**Top H-bond Acceptors**	**Functional group definition**	**Frequency of Occurrence (%)**	**# Structures**	**Coformer acceptor matches**
ar_oh	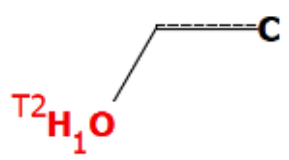	ar_NsingleO	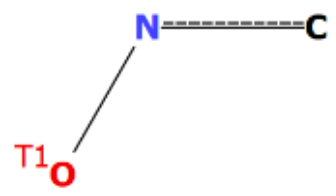	80.18	89	
		ar_PO	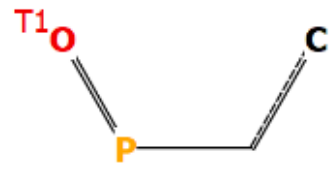	77.53	69	
		Br-	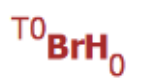	75.89	255	

	cl-	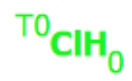	71.83	510
	aromatic_nitrogen	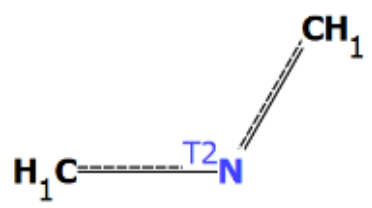	71.57	861
	carboxylate_2	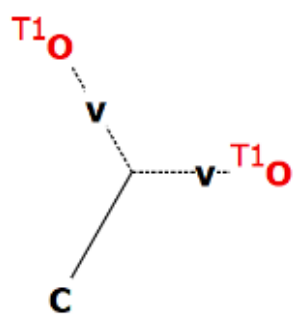	66.73	357
	I-	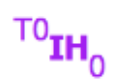	60.0	69
	sulfinyl_2	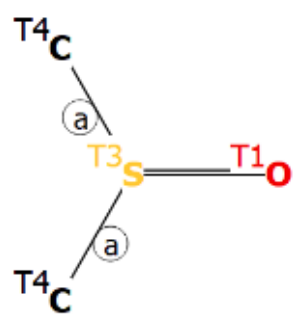	58.58	157

	saturated_ring_NH	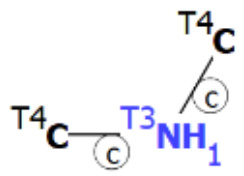	52.21	71	AHEPUY, MUPPUI
	water	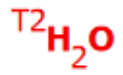	51.99	1816	
	ethanol	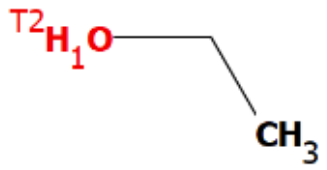	49.86	178	
	methanol	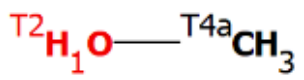	48.37	460	
	ar_CsingleO	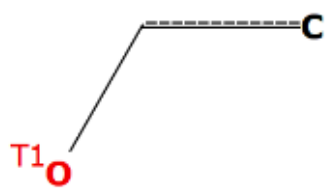	47.59	217	

	ar_nitrogen_2	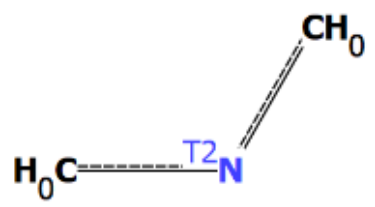	46.85	297	LUJSOZ
	acetone	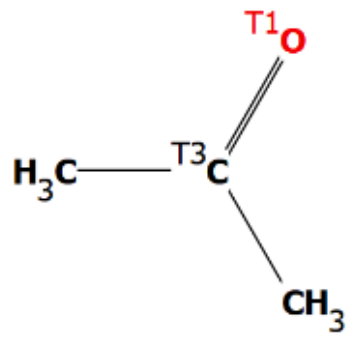	45.39	128	
	ethylacetate	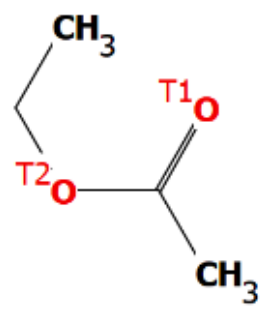	45.22	71	
	THF	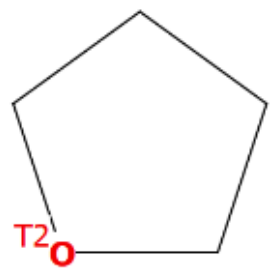	44.92	53	
	dimethylformamide	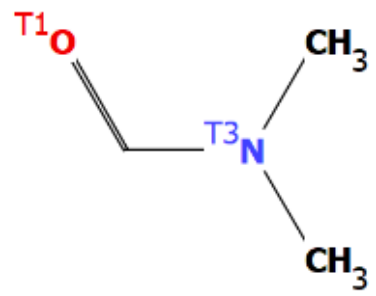	42.97	110	

		cyclic_amide_6	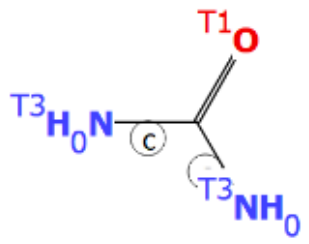	41.36	79	KIGLUI
		ar_sulfonato	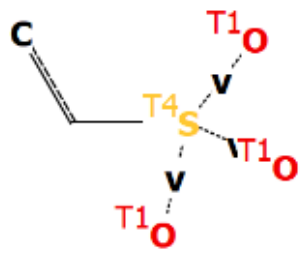	41.25	231	
		imidazole_3	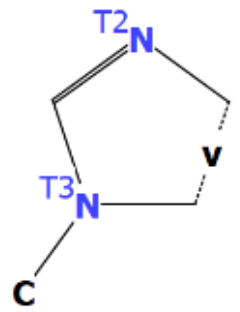	36.7	69	
		ar_N_2	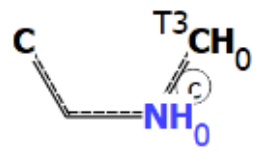	35.7	206	
		al_hydroxy_1	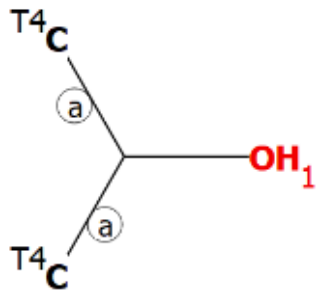	35.37	58	

	al_tert_amine_1	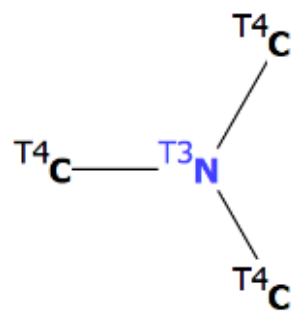	34.04	145	MUPPIW
	cyclic_ketone_1	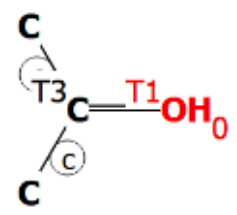	33.82	276	
	ar_carboxylate	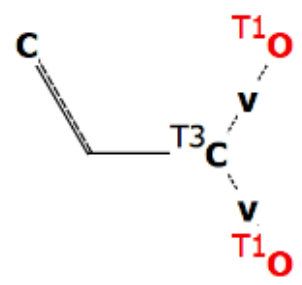	32.62	365	SUTVAF
	carbonyl_2	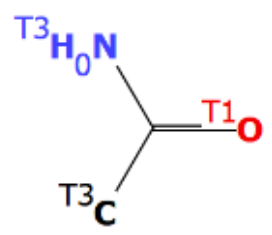	30.64	129	KIGLUI
	acyclic_al_oh	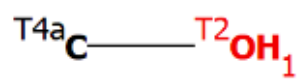	28.05	145	AMUBAM

ar_al_trans_amide	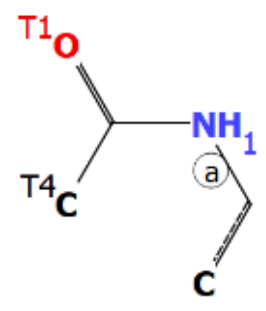	carbamoyl_2	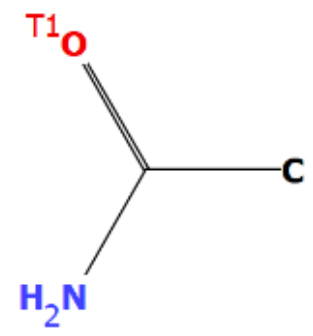	28.04	106
		cyclic_amide_4	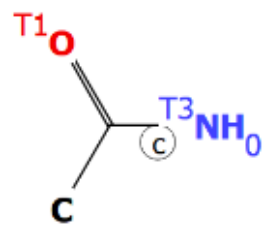	27.08	52
		cl-	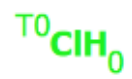	79.41	54
		water	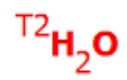	50.91	140
		ar_al_trans_amide	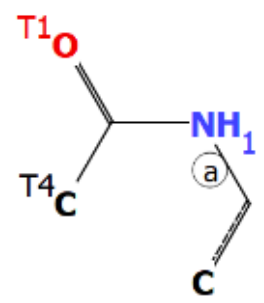	15.93	137
		NA	NA	NA	NA
		NA	NA	NA	NA
		NA	NA	NA	NA

		NA	NA	NA	NA
		NA	NA	NA	NA
		NA	NA	NA	NA
		NA	NA	NA	NA
		NA	NA	NA	NA
		NA	NA	NA	NA
		NA	NA	NA	NA
		NA	NA	NA	NA
		NA	NA	NA	NA
		NA	NA	NA	NA
		NA	NA	NA	NA
		NA	NA	NA	NA
		NA	NA	NA	NA
		NA	NA	NA	NA
		NA	NA	NA	NA
		NA	NA	NA	NA
		NA	NA	NA	NA
		NA	NA	NA	NA
		NA	NA	NA	NA
		NA	NA	NA	NA
		NA	NA	NA	NA
		NA	NA	NA	NA
		NA	NA	NA	NA
		NA	NA	NA	NA

Table 2.3. Top 30 interactions per donor in HXACAN by frequency of occurrence for single component database hits (NCE = 1, ≥ 10 structure threshold), and acceptor matches found in the specified coformer library.

		**Single component (NCE = 1)**				
**H-bond Donor in HXACAN**	**Functional group definition**	**Top H-bond Acceptors**	**Functional group definition**	**Frequency of Occurrence (%)**	**# Structures**	**Coformer acceptor matches**
ar_oh	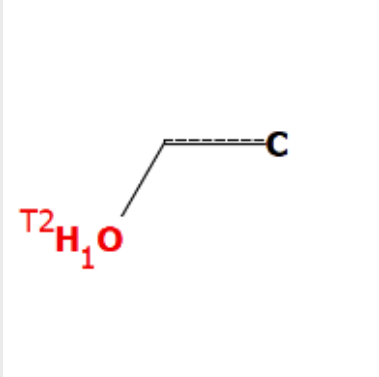	ar_sulfonato	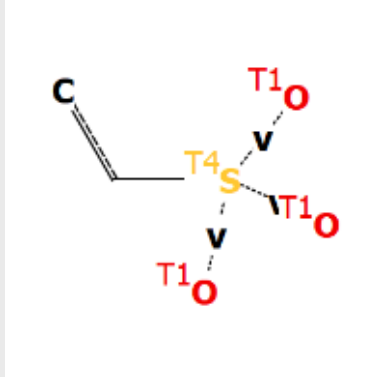	85.71	12	
		carboxylate_2	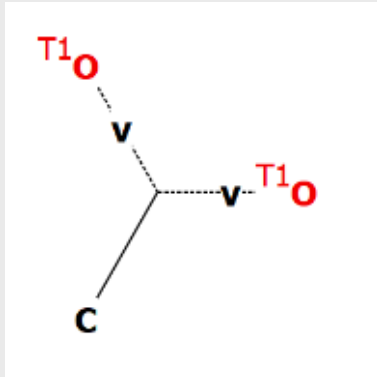	83.33	45	
		cyclic_NH_urea	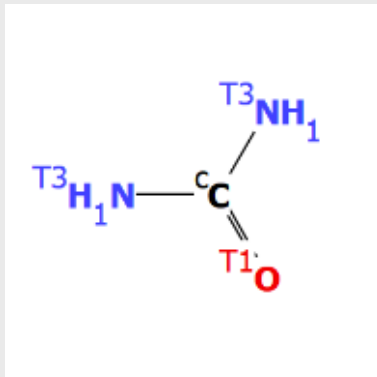	76.19	16	
		pyrazolone_1	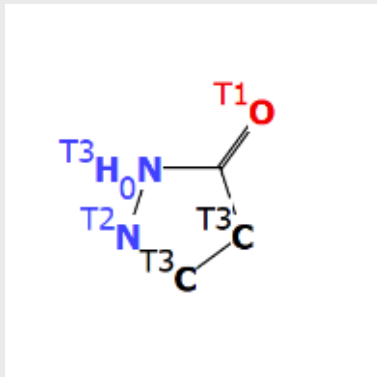	68.75	11	

		ar_CsingleO	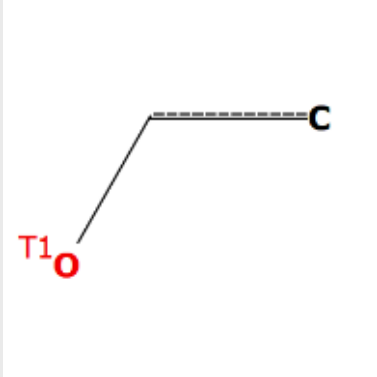	68.18	120	
		phosphonate	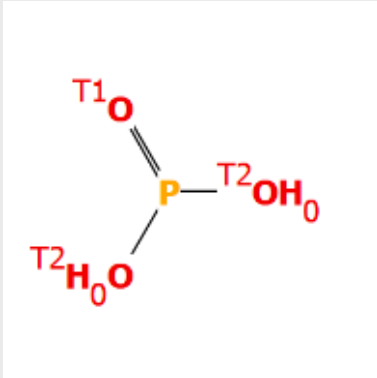	67.23	80	
		sulfinyl_1	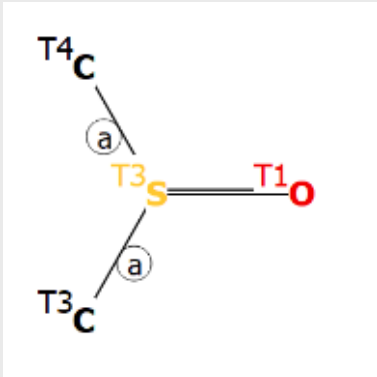	64.71	11	
		al_prim_amine	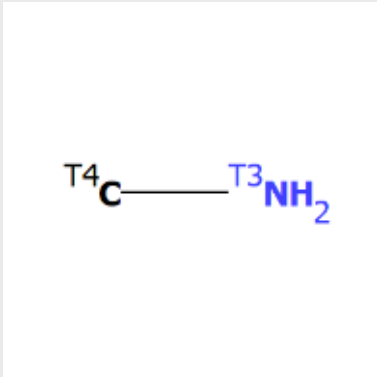	61.29	19	WIGCEW
		imidazole_3	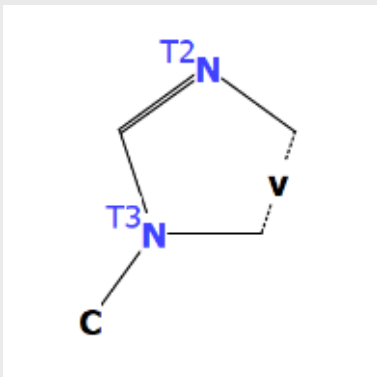	60.0	21	

		CH_aldehyde	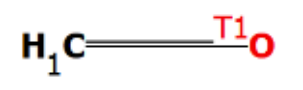	59.09	26
		ar_PO	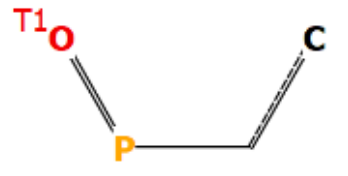	58.7	81
		ar_al_sulfone	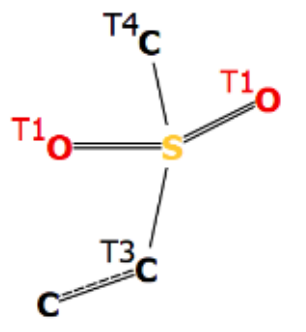	57.5	23
		saturated_ring_amide	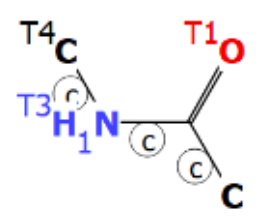	56.72	38
		cyclic_NsingleN	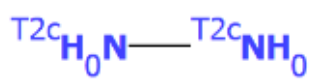	55.74	34

		acyclic_tert_amide	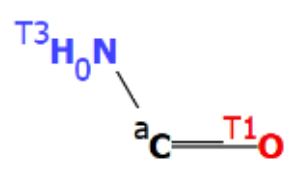	55.26	21
		acyclic_NsingleO	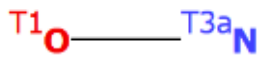	53.57	15
		ar_al_trans_amide	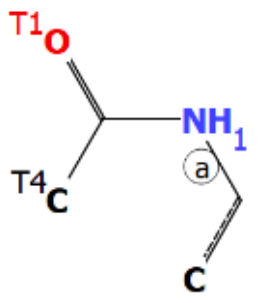	51.47	70
		triazole_1	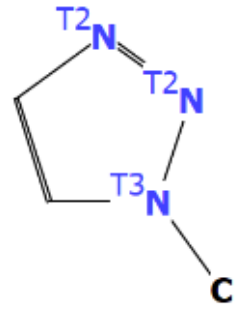	51.43	36
		al_carbonyl_1	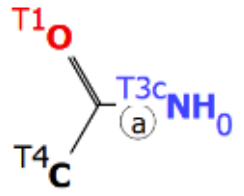	51.32	39

		NH1_thioamide	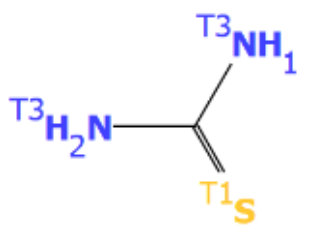	50.0	17	
		gamma_lactone	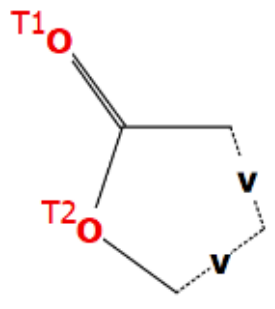	48.65	126	
		al_al_amide	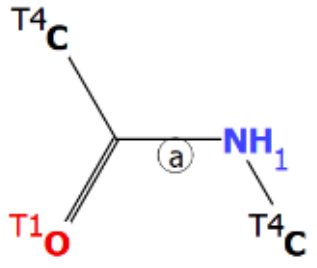	48.54	83	
		carbonyl_2	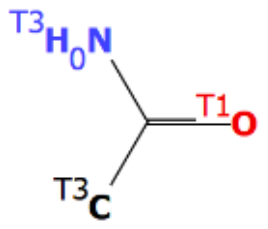	47.97	260	KIGLUI
		ar_ar_amide_1	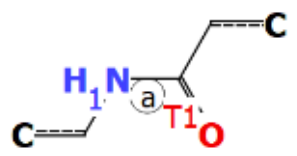	47.79	54	

		ar_sulfonyl	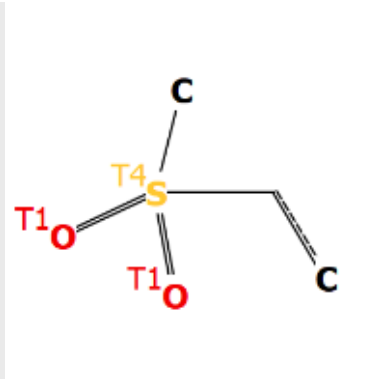	46.58	34	
		al_hydroxy_2	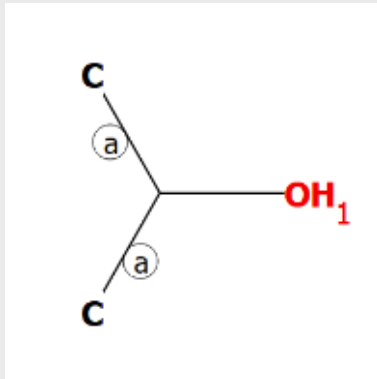	46.34	57	
		acyclic_NH_thiourea	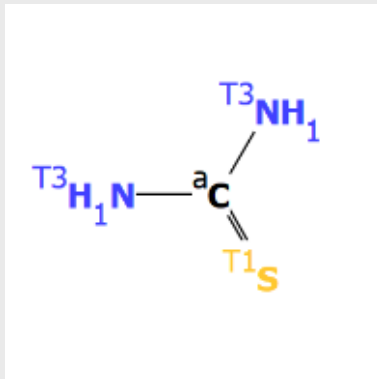	45.38	59	
		cyclic_amide_6	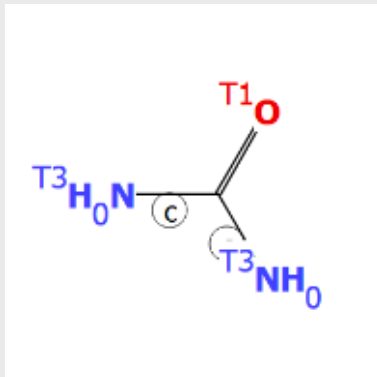	44.83	13	KIGLUI
		acyclic_al_oh	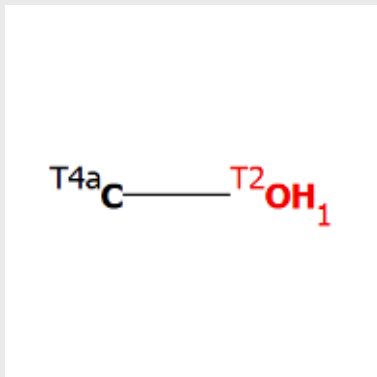	43.92	231	AMUBAM

ar_al_trans_amide	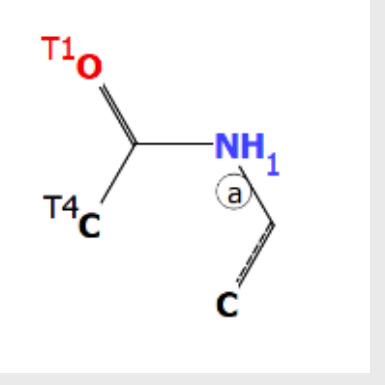	cyclic_amide_4	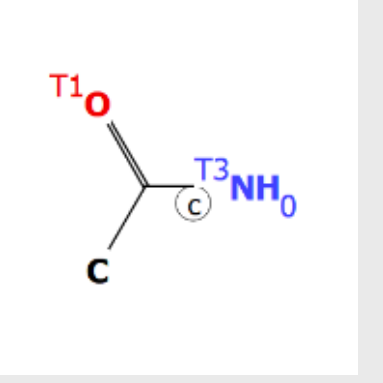	43.7	111
		phosphonate	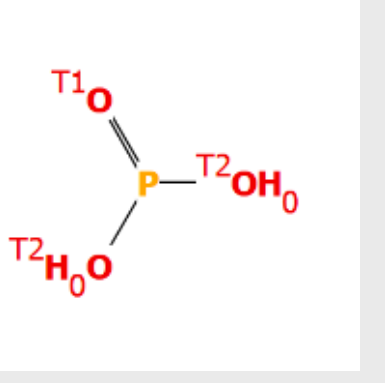	76.47	13
		cyclic_NsingleN	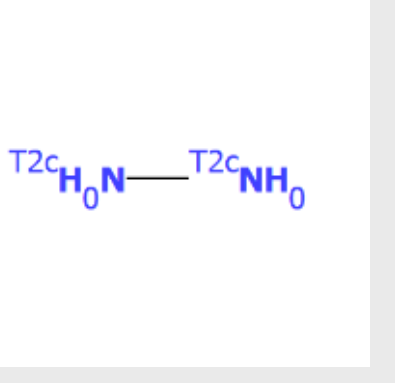	61.9	13
		al_hydroxy_1	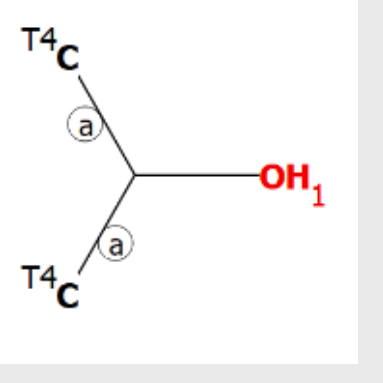	55.0	11
		ar_al_sulfone	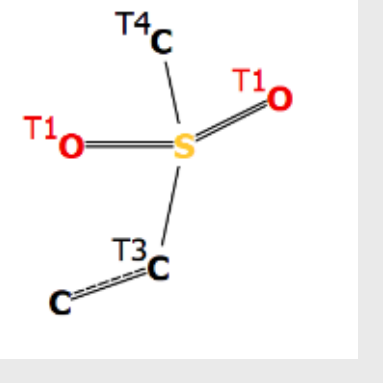	50.0	10

		aromatic_nitrogen	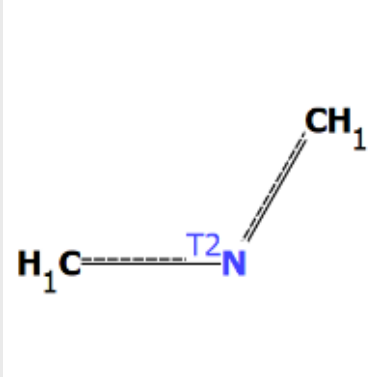	42.19	27	
		ar_al_trans_amide	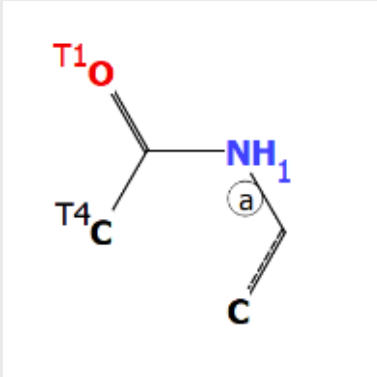	41.84	1235	
		cyclic_amide_4	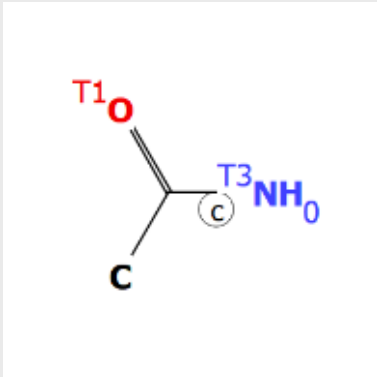	39.74	31	
		sulfonamide_1	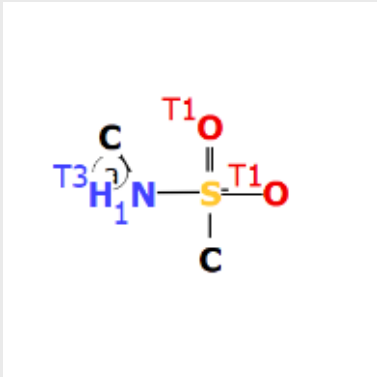	35.0	28	
		carbonyl_2	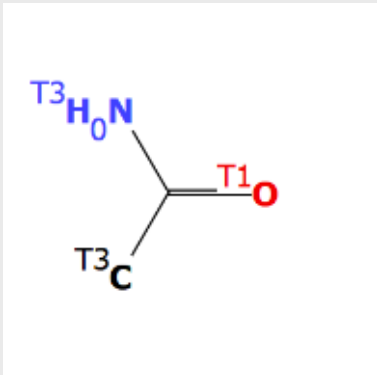	34.71	42	KIGLUI

		cyano	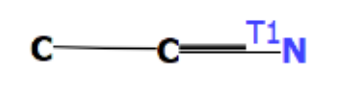	32.69	17	RECJOC
		cyclic_ketone_1	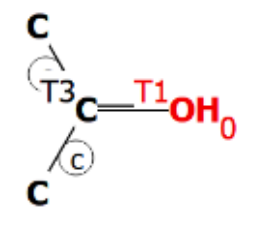	27.91	12	
		ar_ar_amide_1	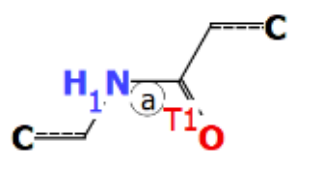	27.78	10	
		al_hydroxy_3	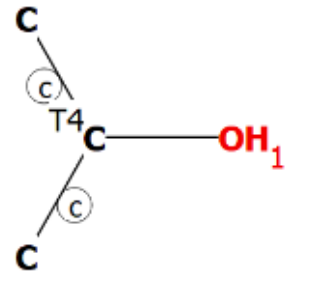	26.32	10	
		amide_carbonyl	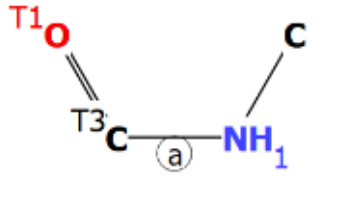	26.09	24	

		al_hydroxy_2	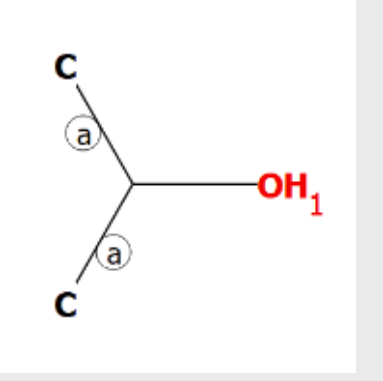	25.0	11	
		al_carbonyl_1	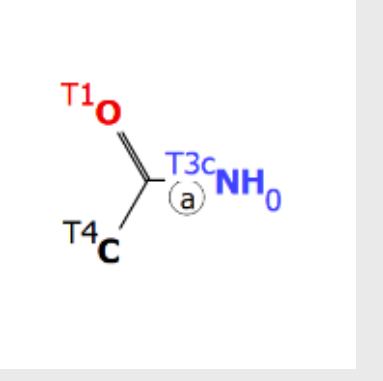	24.39	10	
		R2N_COOR	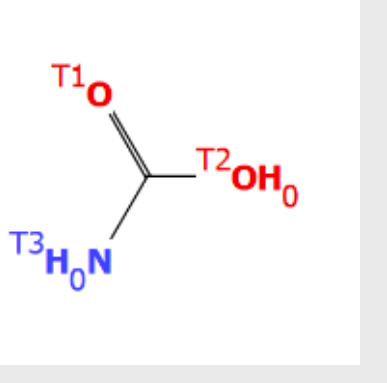	24.0	18	
		acyclic_al_oh	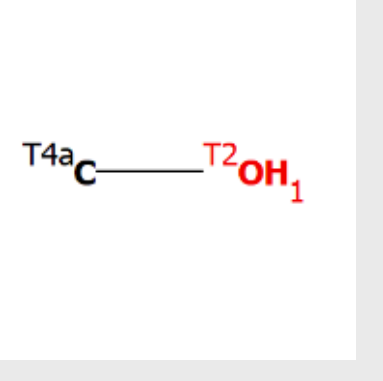	22.81	13	AMUBAM
		al_al_amide	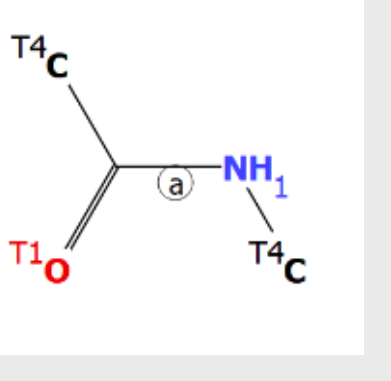	19.05	16	

		ar_oh	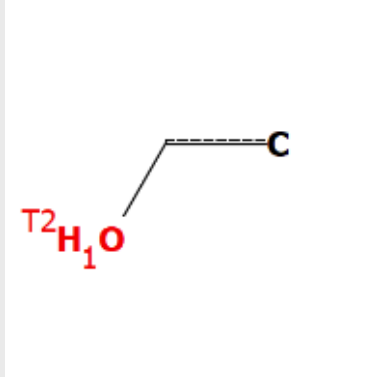	18.38	25	BODSUL
		acetyl_1	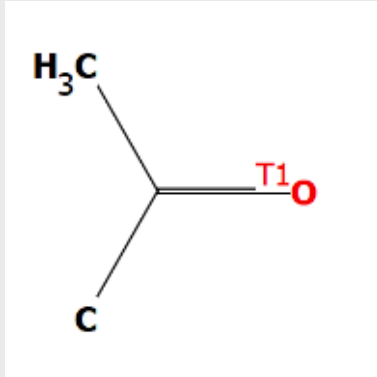	17.2	16	
		al_cooh_1	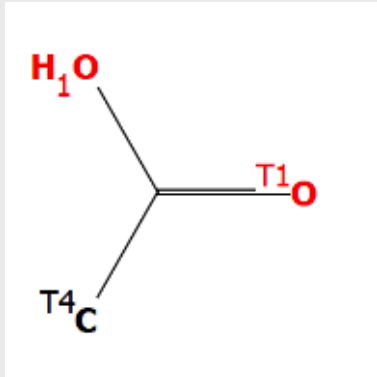	17.07	14	AMUBAM
		ar_nitro	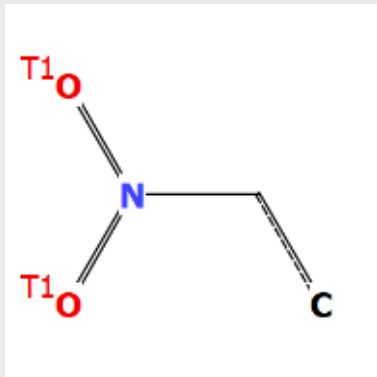	14.61	32	BODSUL
		ar_nitrogen_2	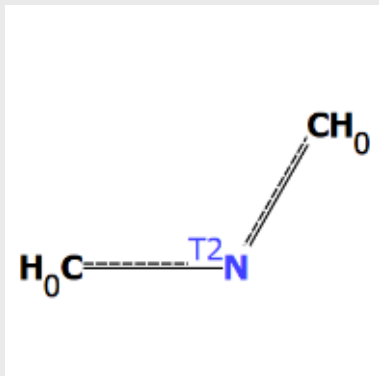	12.7	16	LUJSOZ

		ar_al_ester	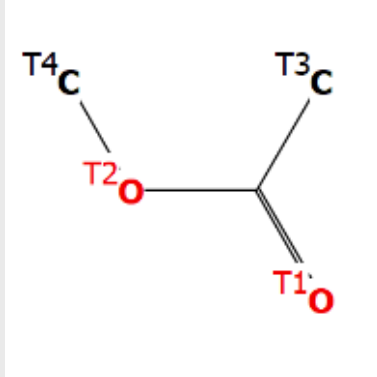	10.14	15
		acyclic_NH1_COOR	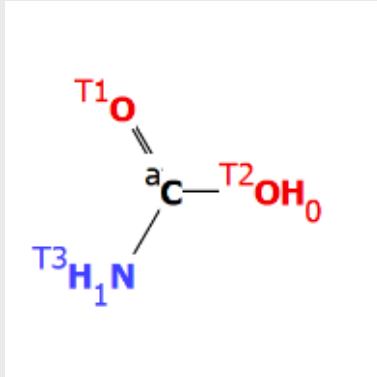	8.4	10
		ar_N_2	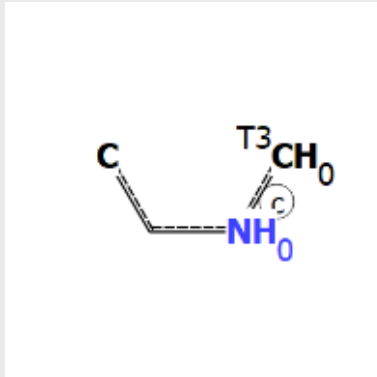	7.49	26
		ar_methoxy	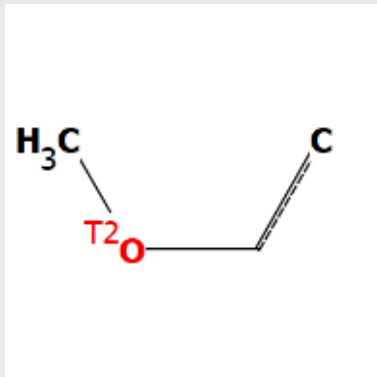	5.86	15
		NA	NA	NA	NA
		NA	NA	NA	NA

Table 2.4. Coformers with top ranked acceptors consolidated across individual donors in HXACAN for multicomponent database hits (NCE > 1).

Table 2.5. Coformers with top ranked acceptors consolidated across individual donors in HXACAN for single component database hits (NCE = 1).

Donor in HXACAN	Coformer	# Acceptor matches in top 30 interactions found in multicomponent structures
ar_oh	KIGLUI	2
ar_oh	AHEPUY	1
ar_oh	MUPPUI	1
ar_oh	LUJSOZ	1
ar_oh	MUPPIW	1
ar_oh	SUTVAF	1
ar_oh	AMUBAM	1

Donor in HXACAN	Coformer	# Acceptor matches in top 30 interactions found in single component structures
ar_oh	KIGLUI	2
ar_oh	WIGCEW	1
ar_oh	AMUBAM	1
ar_al_trans_amide	AMUBAM	2
ar_al_trans_amide	BODSUL	2
ar_al_trans_amide	KIGLUI	1
ar_al_trans_amide	RECJOC	1
ar_al_trans_amide	LUJSOZ	1

Table 2.6. Coformers with top ranked acceptors consolidated across all donors in HXACAN for NCE > 1.

Coformer	# Acceptor matches in top 30 interactions found in multicomponent structures
KIGLUI	2
AHEPUY	1

Table 2.7. Coformers with top ranked acceptors consolidated across all donors in HXACAN for NCE = 1.

Coformer	# Acceptor matches in top 30 interactions found in single component structures
KIGLUI	3
AMUBAM	3
BODSUL	2
WIGCEW	1

MUPPUI	1
LUJSOZ	1
MUPPIW	1
SUTVAF	1
AMUBAM	1

RECJOC	1
LUJSOZ	1

Top 10 interactions per acceptor in HXACAN

Table 2.8. Top 30 interactions per acceptor in HXACAN by frequency of occurrence for multicomponent database hits (NCE > 1, ≥ 50 structure threshold), and donor matches found in the specified coformer library.

		**Multicomponent (NCE > 1)**				
**H-bond Acceptor in HXACAN**	**Functional group definition**	**Top H-Bond Donors**	**Functional group definition**	**Frequency of Occurrence (%)**	**# Structures**	**Coformer donor matches**
ar_oh	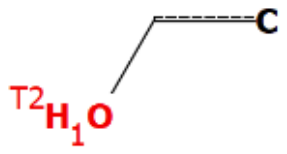	ar_prim_amine	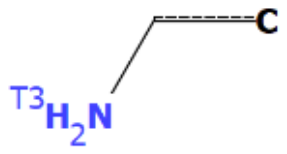	52.66	168	
		water	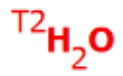	38.59	1348	
		carbamoyl_2	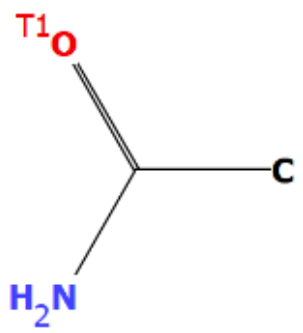	33.07	125	

	acyclic_T4C_nh3	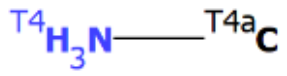	31.92	128	
	ethanol	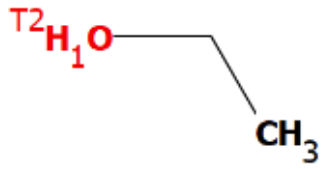	22.69	81	
	methanol	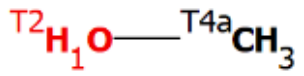	21.98	209	
	al_hydroxy_3	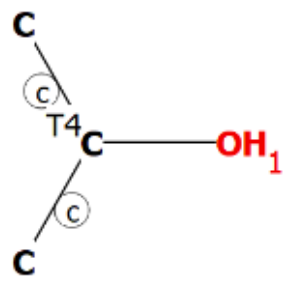	19.35	154	
	acyclic_al_oh	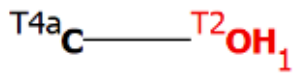	18.57	96	AMUBAM

ar_oh	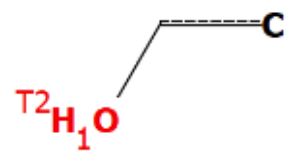	12.09	1478	BODSUL
NA	NA	NA	NA	
NA	NA	NA	NA	
NA	NA	NA	NA	
NA	NA	NA	NA	
NA	NA	NA	NA	
NA	NA	NA	NA	
NA	NA	NA	NA	
NA	NA	NA	NA	
NA	NA	NA	NA	
NA	NA	NA	NA	
NA	NA	NA	NA	
NA	NA	NA	NA	
NA	NA	NA	NA	
NA	NA	NA	NA	
NA	NA	NA	NA	
NA	NA	NA	NA	
NA	NA	NA	NA	
NA	NA	NA	NA	
NA	NA	NA	NA	
NA	NA	NA	NA	
NA	NA	NA	NA	

ar_al_trans_amide	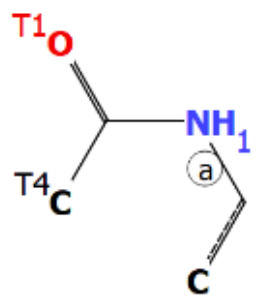	water	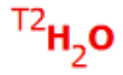	52.36	144
		ar_al_trans_amide	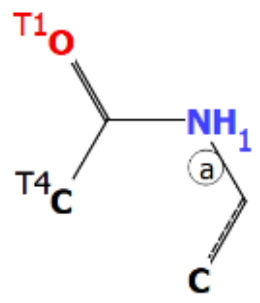	15.93	137
		NA	NA	NA	NA
		NA	NA	NA	NA
		NA	NA	NA	NA
		NA	NA	NA	NA
		NA	NA	NA	NA
		NA	NA	NA	NA
		NA	NA	NA	NA
		NA	NA	NA	NA
		NA	NA	NA	NA
		NA	NA	NA	NA
		NA	NA	NA	NA
		NA	NA	NA	NA
		NA	NA	NA	NA
		NA	NA	NA	NA
		NA	NA	NA	NA
		NA	NA	NA	NA
		NA	NA	NA	NA

		NA	NA	NA	NA	
		NA	NA	NA	NA	
		NA	NA	NA	NA	
		NA	NA	NA	NA	
		NA	NA	NA	NA	
		NA	NA	NA	NA	
		NA	NA	NA	NA	
		NA	NA	NA	NA	
		NA	NA	NA	NA	
		NA	NA	NA	NA	
		NA	NA	NA	NA	

Table 2.9. Top 30 interactions per acceptor in HXACAN by frequency of occurrence for single component database hits (NCE = 1, ≥ 10 structure threshold), and donor matches found in the specified coformer library.

		**Single component (NCE = 1)**				
**H-bond Acceptor in HXACAN**	**Functional group definition**	**Top H-Bond Donors**	**Functional group definition**	**Frequency of Occurrence (%)**	**# Structures**	**Coformer donor matches**
ar_oh	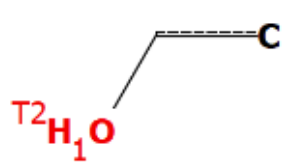	acyclic_nh_prim_amine	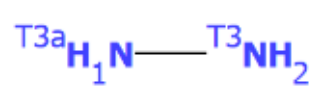	85.71	12	

		hydroxyimino	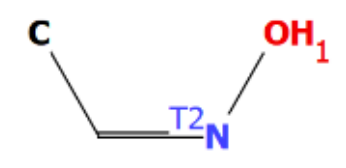	78.43	40	
		NH1_thioamide	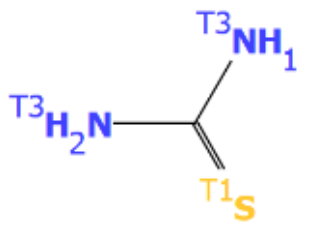	67.65	23	
		ar_prim_amine	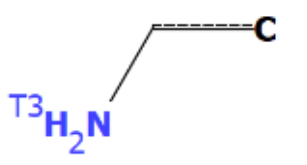	60.36	102	
		ar_N_NH2	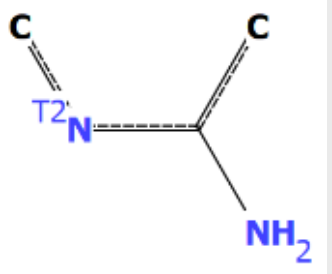	50.0	10	
		al_prim_amine	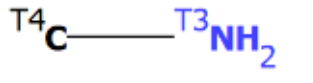	48.39	15	WIGCEW

		acyclic_T4C_nh3	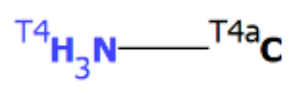	48.15	13
		acyclic_prim_amine	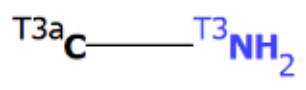	46.67	21
		cyclic_prim_amine	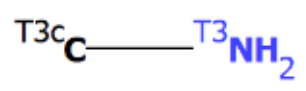	46.3	25
		pyrazoline_1	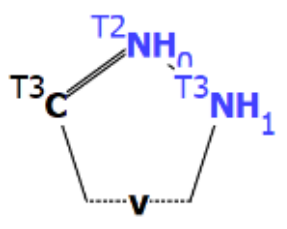	45.71	32
		coordinated_water	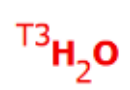	42.31	11

		carbamoyl_2	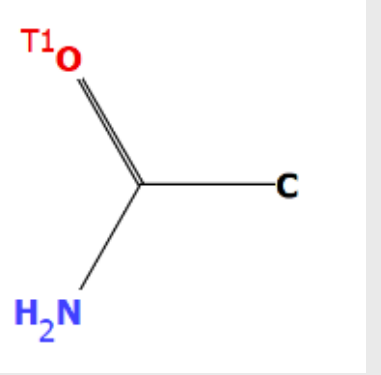	42.25	30
		acyclic_NH_thiourea	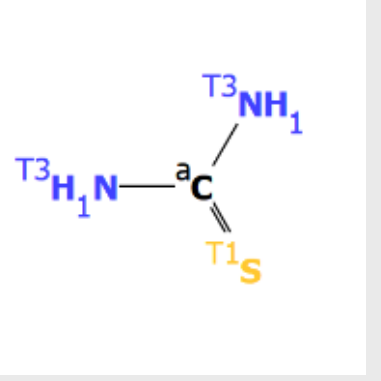	40.0	52
		indole	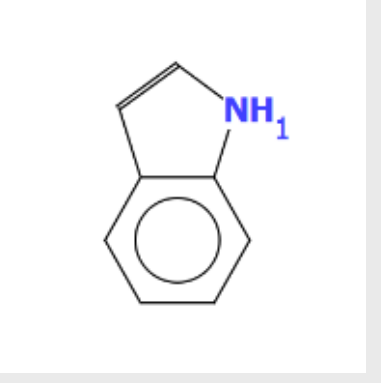	40.0	50
		imidazole_1	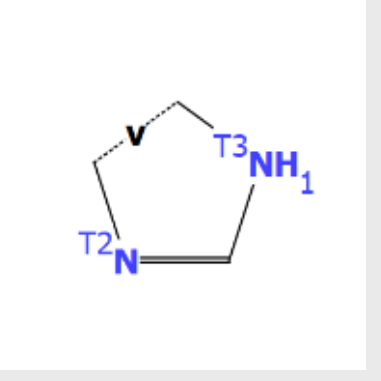	39.56	36
		hydroxyimino_2	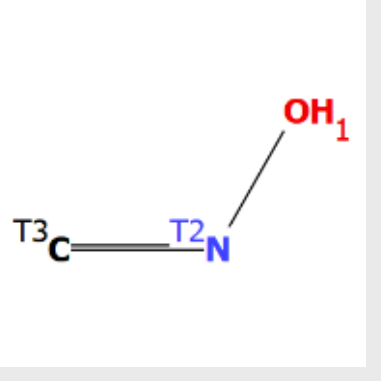	35.9	14

		al_hydroxy_6	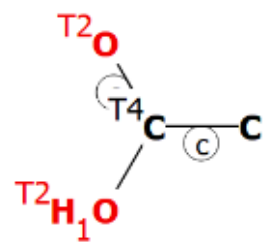	32.31	21	
		ar_al_amine	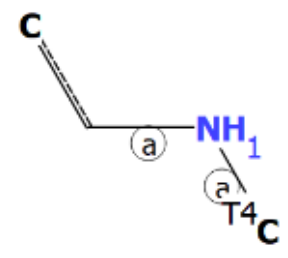	32.0	48	
		acyclic_al_oh	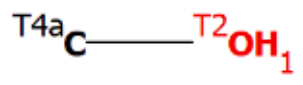	30.23	159	AMUBAM
		acyclic_NH_urea	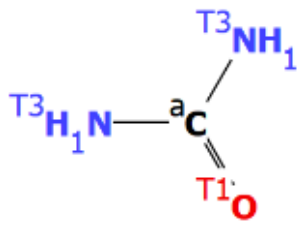	30.23	13	
		amide_1	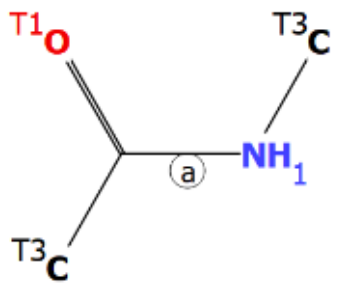	25.81	16	

		al_hydroxy_2	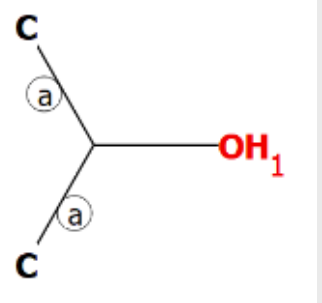	25.2	31	
		saturated_ring_NH	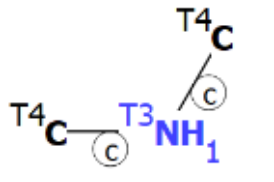	24.64	17	AHEPUY, MUPPUI
		amide_carbonyl	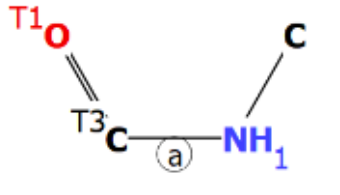	24.18	37	
		al_hydroxy_3	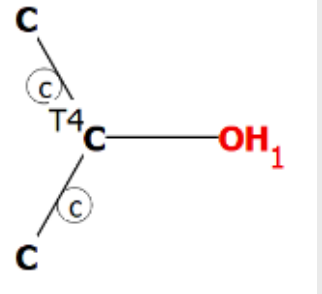	24.1	160	
		sec_amine_pl	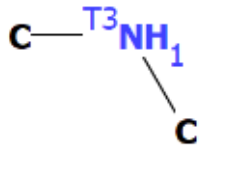	23.49	74	

		ar_ar_sec_amine	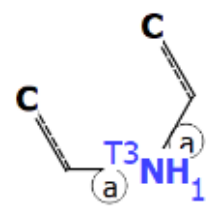	22.22	16
		acyclic_nhn	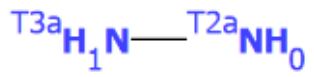	18.98	26
		al_hydroxy_1	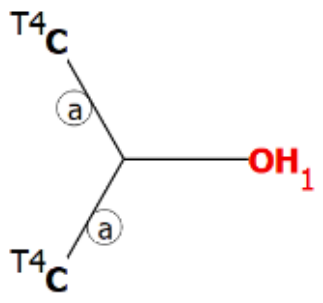	18.81	19
		ar_al_trans_amide	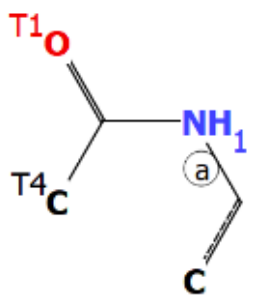	18.38	25
ar_al_trans_amide	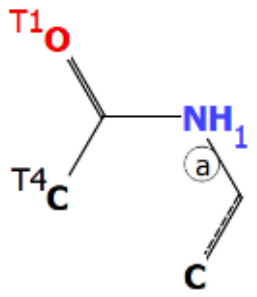	ar_N_NH2	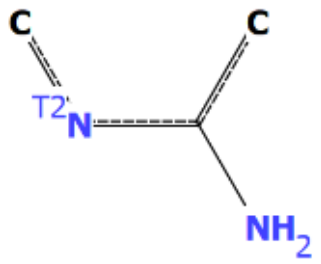	92.86	13

		al_hydroxy_2	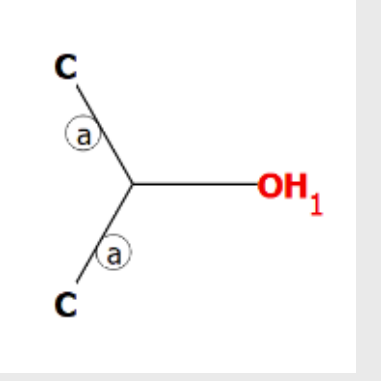	79.55	35
		ar_prim_amine	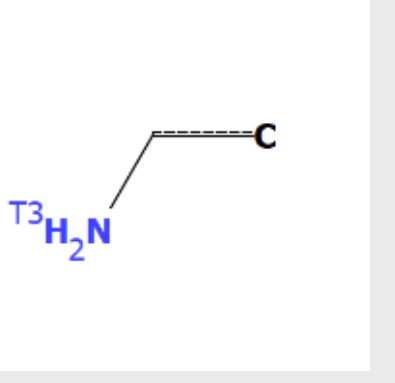	68.18	15
		indole	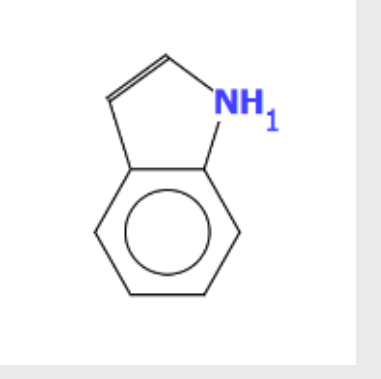	63.64	14
		al_al_sec_amine_1	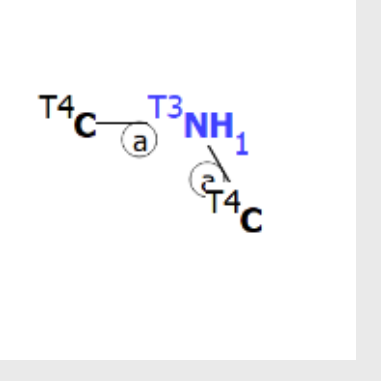	62.07	18
		al_hydroxy_1	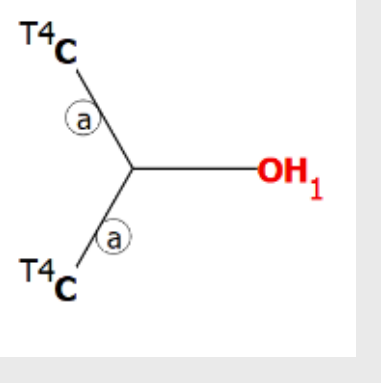	60.0	12

		ar_oh	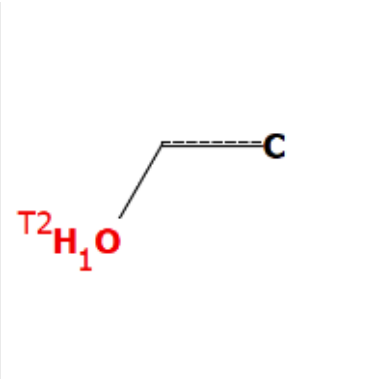	51.47	70	BODSUL
		acyclic_al_oh	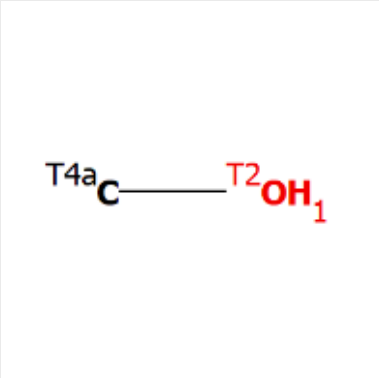	49.12	28	AMUBAM
		sec_amine_pl	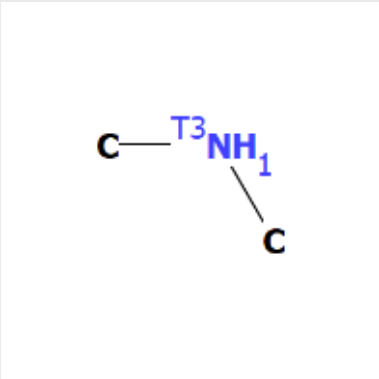	47.06	16	
		ar_cooh_1	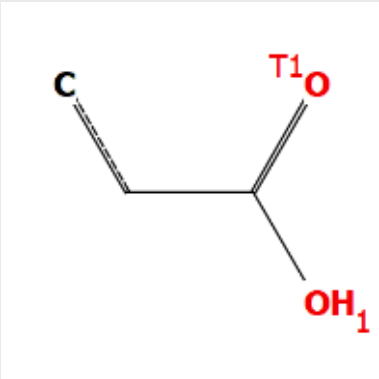	43.48	20	SUTVAF
		al_hydroxy_3	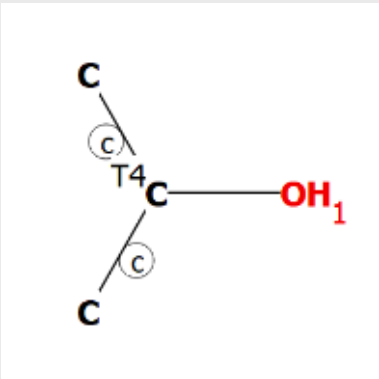	42.11	16	

		ar_al_trans_amide	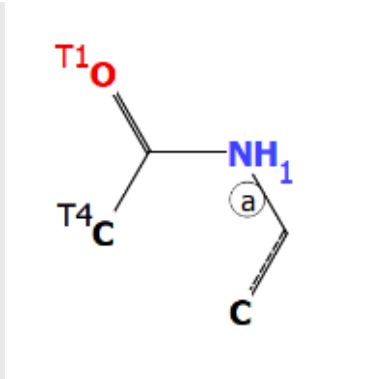	41.84	1235
		amide_carbonyl	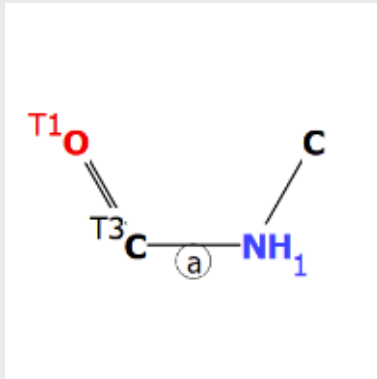	40.22	37
		al_al_amide	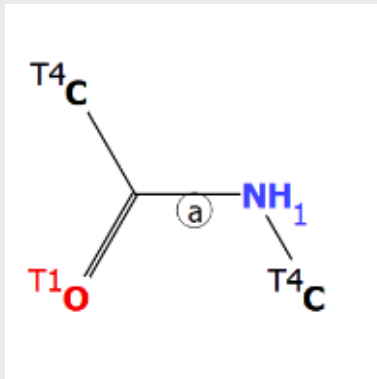	39.29	33
		ar_al_amine	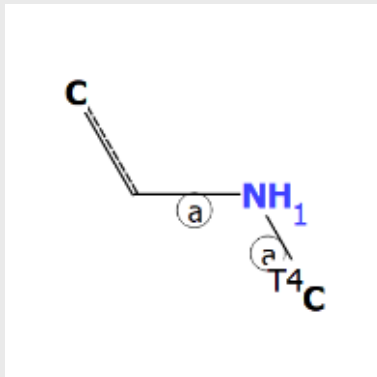	38.24	13
		acyclic_NH1_COOR	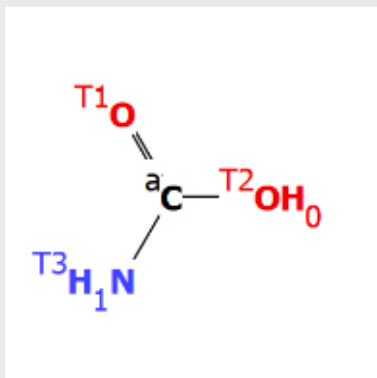	36.97	44

		sulfonamide_1	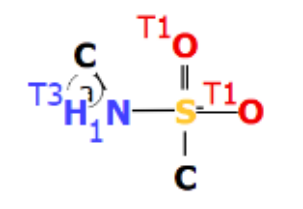	31.25	25	
		al_cooh_1	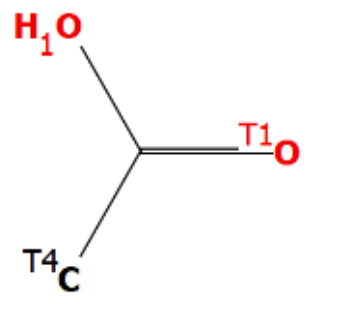	24.39	20	AMUBAM
		NA	NA	NA	NA	
		NA	NA	NA	NA	
		NA	NA	NA	NA	
		NA	NA	NA	NA	
		NA	NA	NA	NA	
		NA	NA	NA	NA	
		NA	NA	NA	NA	
		NA	NA	NA	NA	
		NA	NA	NA	NA	
		NA	NA	NA	NA	
		NA	NA	NA	NA	
		NA	NA	NA	NA	

Table 2.10. Coformers with top ranked donors consolidated across individual acceptors in HXACAN for multicomponent database hits (NCE > 1).

Acceptor in HXACAN	Coformer	# Donor matches in top 30 interactions found in multicomponent structures
ar_oh	AMUBAM	1
ar_oh	BODSUL	1

Table 2.11. Coformers with top ranked donors consolidated across individual acceptors in HXACAN for single component database hits (NCE = 1).

Acceptors in HXACAN	Coformer	# Donor matches in top 30 interactions found in single component structures
ar_oh	WIGCEW	1
ar_oh	AMUBAM	1
ar_oh	AHEPUY	1
ar_oh	MUPPUI	1
ar_al_trans_amide	AMUBAM	2
ar_al_trans_amide	BODSUL	1
ar_al_trans_amide	SUTVAF	1

Table 2.12. Coformers with top ranked donors consolidated across all acceptors in HXACAN for NCE > 1.

Coformer	# Donor matches in top 30 interactions

Table 2.13. Coformers with top ranked donors consolidated across all acceptors in HXACAN for NCE = 1.

Coformer	# Donor matches in top 30 interactions

	**found in multicomponent structures**
AMUBAM	1
BODSUL	1

	**found in single component structures**
AMUBAM	3
WIGCEW	1
AHEPUY	1
MUPPUI	1
BODSUL	1
SUTVAF	1

Functional groups identified in HXACAN and the coformers

Table 2.14. List of functional groups in HXACAN.

Atom label	Donors
N1	ar_al_trans_amide
O1	ar_oh
**Atom label**	**Acceptors**
O1	ar_oh
O2	ar_al_trans_amide

Table 2.15. List of functional groups in the coformer library.

Coformer	Donors
AHEPUY	['saturated_ring_NH']
AMUBAM	['acyclic_al_oh', 'al_cooh_1']
BODSUL	['ar_oh']
JEMTEE	['carboxylic_acid']
KIGLUI	['imidazole_2']
LUJTAM	['keto_carboxylic_acid']
MUPPUI	['saturated_ring_NH']
SUTVAF	['ar_cooh_1', 'ar_nh']
WIGCEW	['al_prim_amine']
**Atom label**	**Acceptors**
AHEPUY	['saturated_ring_NH', 'cyclic_ether_1']
AMUBAM	['acyclic_al_oh', 'al_cooh_1']
BODSUL	['ar_nitro', 'ar_oh']
JEMTEE	['carboxylic_acid']
KIGLUI	['cyclic_amide_6', 'carbonyl_2', 'imidazole_2']
LUJSOZ	['ar_nitrogen_2']
LUJTAM	['keto_carboxylic_acid']
MUPPES	['dioxane']
MUPPIW	['al_tert_amine_1']
MUPPUI	['saturated_ring_NH']
RECJOC	['cyano']
SUTVAF	['ar_carboxylate', 'ar_cooh_1']
WIGCEW	['al_prim_amine']
